# Polarizable atomic multipole X-ray refinement: application to peptide crystals

**DOI:** 10.1107/S0907444909022707

**Published:** 2009-08-14

**Authors:** Michael J. Schnieders, Timothy D. Fenn, Vijay S. Pande, Axel T. Brunger

**Affiliations:** a Department of Chemistry, Stanford, CA 94305, USA; b Department of Molecular and Cellular Physiology, Stanford, CA 94305, USA; c Howard Hughes Medical Institute, USA

**Keywords:** scattering factors, aspherical, anisotropic, force fields, multipole, polarization, AMOEBA, bond density, direct summation, FFT, SGFFT, Ewald, PME

## Abstract

Recent advances in computational chemistry have produced force fields based on a polarizable atomic multipole description of biomolecular electrostatics. In this work, the Atomic Multipole Optimized Energetics for Biomolecular Applications (AMOEBA) force field is applied to restrained refinement of molecular models against X-ray diffraction data from peptide crystals. A new formalism is also developed to compute anisotropic and aspherical structure factors using fast Fourier transformation (FFT) of Cartesian Gaussian multipoles. Relative to direct summation, the FFT approach can give a speedup of more than an order of magnitude for aspherical refinement of ultrahigh-resolution data sets. Use of a sublattice formalism makes the method highly parallelizable. Application of the Cartesian Gaussian multipole scattering model to a series of four peptide crystals using multipole coefficients from the AMOEBA force field demonstrates that AMOEBA systematically underestimates electron density at bond centers. For the trigonal and tetrahedral bonding geometries common in organic chemistry, an atomic multipole expansion through hexadecapole order is required to explain bond electron density. Alternatively, the addition of inter­atomic scattering (IAS) sites to the AMOEBA-based density captured bonding effects with fewer parameters. For a series of four peptide crystals, the AMOEBA–IAS model lowered *R*_free_ by 20–40% relative to the original spherically symmetric scattering model.

## Abstract 2

A method to accelerate the computation of structure factors from an electron density described by anisotropic and aspherical atomic form factors *via* fast Fourier transformation is described for the first time.

## 1. Introduction

The number of X-ray crystal structures in the Protein Data Bank (PDB) with a resolution of higher than 1.0 Å continues to increase rapidly (Berman *et al.*, 2000 ▶). In late 2002, there were already over 100 structures available at subatomic resolution (Afonine & Urzhumtsev, 2004 ▶), while as of early 2009 the number had more than tripled to well over 300. Examples include the proteins lysozyme at 0.65 Å (Wang *et al.*, 2007 ▶), aldose reductase at 0.66 Å (Howard *et al.*, 2004 ▶) and serine protease at 0.78 Å (Kuhn *et al.*, 1998 ▶), as well as nucleic acid structures such as B-DNA at 0.74 Å (Kielkopf *et al.*, 2000 ▶), Z-DNA at 0.60 Å (Tereshko *et al.*, 2001 ▶) and an RNA tetraplex at 0.61 Å (Deng *et al.*, 2001 ▶). Crystals that diffract to high resolution are ideal for studying valence-electron distributions (Jelsch *et al.*, 2000 ▶; Muzet *et al.*, 2003 ▶; Zarychta *et al.*, 2007 ▶; Volkov *et al.*, 2007 ▶; Coppens & Volkov, 2004 ▶) that dictate the electrostatic properties of macromolecules. Electrostatics, in turn, is one of the driving forces in protein and nucleic acid folding, which should be understood in detail in order to predict biomolecular thermodynamics and kinetics (Snow *et al.*, 2002 ▶, 2005 ▶; Sorin & Pande, 2005 ▶; Pande *et al.*, 2003 ▶). In this work, we contribute an improved theory and algorithm for computing the anisotropic and aspherical valence-electron density of molecules for X-ray crystal structure refinement.

Calculation of structure factors is generally based on scattering factors defined by the isolated-atom model (IAM), which assumes that the electron density around each atom is spherically symmetric. However, subatomic resolution diffraction data capture aspherical features of the electron density that result from bonding and the local chemical environment. The difference between the IAM and the true electron density is defined as the deformation density. For example, aspherical electron-density models of diamond, silicon and germanium developed by DeMarco and Weiss and later by Dawson explained the peaks of deformation density at bond midpoints observed in the experimental data (Dawson, 1967*a* ▶,*b* ▶,*c* ▶; DeMarco & Weiss, 1965 ▶). In these works, the IAM was augmented by atom-centered spherical harmonic expansions, whose physical consequence was to redistribute electron density from nonbonding lobes into the tetragonal arrangement of bond centers.

A variety of radial functions have been used in combination with atom-centered spherical harmonic expansions. Modified Gaussians were promoted by Dawson (1967*a* ▶), a set of harmonic oscillator wavefunctions by Kurki-Suonio (1968 ▶) and more recently a formalism based on Slater-type orbitals (STO) was described by Stewart and coworkers (Epstein *et al.*, 1977 ▶; Cromer *et al.*, 1976 ▶; Stewart, 1979 ▶, 1977 ▶) and by Hansen & Coppens (1978 ▶), which represents the current standard (Jelsch *et al.*, 2005 ▶; Zarychta *et al.*, 2007 ▶; Volkov *et al.*, 2007 ▶; Coppens, 2005 ▶). However, spherical harmonics are not the only basis set available to describe the angular dependence of the deformation density.

We first present a formulation of anisotropic and aspherical atomic densities based on Cartesian Gaussian multipoles, which leads to much simpler formulae for the calculation of structure factors *via* direct summation in reciprocal space than the STO-based theory of Hansen & Coppens (1978 ▶). We also demonstrate that Cartesian Gaussian multipoles allow the computation of structure factors *via* fast Fourier transformation (FFT) of the real-space electron density (Cooley & Tukey, 1965 ▶). The latter approach, originally proposed by Ten Eyck (1973 ▶, 1977 ▶), is the basis of the efficient macromolecular refinement algorithms (Brünger, 1989 ▶; Afonine & Urzhum­tsev, 2004 ▶; Afonine *et al.*, 2007 ▶; Agarwal, 1978 ▶) implemented in programs such as *CNS* (Brünger *et al.*, 1998 ▶; Brunger, 2007 ▶) and *PHENIX* (Adams *et al.*, 2002 ▶). The sublattice method implemented in *CNS* lends itself to efficient parallelization (Brünger, 1989 ▶).

Boys originally proposed Cartesian Gaussian functions as basis functions to solve the many-electron Schrödinger equation (Boys, 1950 ▶). The advantage of Gaussians over STOs in this context is that two-electron integrals have analytic forms, which has led to the adoption of Gaussian basis sets for many *ab initio* calculations (Hehre *et al.*, 1969 ▶, 1970 ▶). We note that the equivalence of spherical harmonics and Cartesian tensors is well known, with key relationships having been presented by Stone (1996 ▶) and Applequist (1989 ▶, 2002 ▶).

We apply Cartesian Gaussian multipoles to restrained crystallographic refinements based on the Atomic Multipole Optimized Energetics for Biomolecular Applications (AMOEBA) force-field electrostatic model (Ponder & Case, 2003 ▶; Ren & Ponder, 2002 ▶, 2003 ▶, 2004 ▶; Schnieders *et al.*, 2007 ▶; Schnieders & Ponder, 2007 ▶). The AMOEBA electrostatic model is based on the superposition of permanent atomic multipoles truncated at quadrupoles and induced dipoles. Permanent electrostatics represents the electron density of a group of atoms in the absence of interactions with the environment, which may include other parts of the molecule or solvent. Groups are chosen to be relatively rigid in order to avoid conformational variability in the permanent multipole moments. Conversely, the induced dipoles of AMOEBA represent polarization, the response of the electron density to the local electric field.

Force fields are widely used to restrain macromolecular refinement by contributing forces to local optimizations and molecular dynamics (Brünger *et al.*, 1987 ▶), with the latter used within simulated-annealing algorithms to promote global optimization (Brünger, 1988 ▶, 1991 ▶; Brünger *et al.*, 1989 ▶, 1990 ▶, 1997 ▶; Kuriyan *et al.*, 1989 ▶; Adams *et al.*, 1997 ▶; Brünger & Rice, 1997 ▶). Up to now, force fields in crystallography have been largely limited to the geometric and repulsive terms and have had no influence on the atomic scattering factors. Therefore, refinement using a scattering model based on AMOEBA electrostatics is novel and lends insight into the progress being made in the development of precise, transferable force fields. Another limitation of the use of force fields for restraining X-­ray refinement has been the lack of proper treatment of long-range electrostatic interactions, which is overcome in this work *via* use of particle-mesh Ewald summation (PME; Darden *et al.*, 1993 ▶; Essmann *et al.*, 1995 ▶; Sagui *et al.*, 2004 ▶).

In addition to AMOEBA, polarizable force fields are being studied by a number of other groups. Maple and coworkers have pursued a model similar to AMOEBA, but with the permanent moments truncated at dipole order, which has shown promising results for protein–ligand complexes (Friesner *et al.*, 2005 ▶; Maple *et al.*, 2005 ▶). As an alternative to induced dipoles, Patel and Brooks employed a fluctuating-charge model of polarization (Patel & Brooks, 2006 ▶), while Lamoureux and Roux have demonstrated success using classical Drude oscillators (Lamoureux *et al.*, 2006 ▶; Lamoureux & Roux, 2003 ▶). In addition to polarization, Gresh and coworkers have developed a methodology to include nonclassical effects such as electrostatic penetration and charge transfer (Gresh, 2006 ▶; Gresh *et al.*, 2007 ▶; Piquemal *et al.*, 2006 ▶, 2007 ▶).

Although classical potentials can be validated against a range of experimental observables, for example small-molecule solvation energies (Shirts *et al.*, 2003 ▶; Shirts & Pande, 2005 ▶), high-resolution diffraction data can pinpoint deficiencies in an electrostatics model with high precision. For example, we show that truncation of permanent atomic multipoles at quadrupole order limits the ability of the AMOEBA model to place charge density at bond midpoints. We use an efficient solution to this limitation by refining partial charges at bond centers as originally proposed by Afonine *et al.* (2007 ▶).

## 2. Theory

### 2.1. Subgrid fast Fourier transform

The starting point for this work is the subgrid fast Fourier transform algorithm (SGFFT), which will be briefly summarized (Brünger, 1989 ▶). In FFT-based methods, the electron density is computed over a lattice chosen to be fine enough to avoid aliasing effects at a given resolution. This computation can be made more efficient by an artificial increase in the atomic displacement parameters (ADPs) of all atoms. The optimum choice in *CNS* v.1.2 (Brunger, 2007 ▶) for the ADP offset and grid size follows the work of Bricogne (2001 ▶). An important point is that the electron density is only computed within a cutoff radius around each atom. As the resolution increases, the cutoff is increased based on an empirical scheme to maintain agreement between direct-summation structure factors and derivatives and the SGFFT calculation (Brunger, 2007 ▶).

Structure factors are computed by FFT of the electron density of an asymmetric unit of atoms (Agarwal, 1978 ▶). The SGFFT is based on factorizing this computation into smaller FFTs that are computed separately on sublattices, which allows efficient parallelization since these tasks are independent (Brünger, 1989 ▶; Kay Diederichs, private communication). *CNS* v.1.21 has implemented this approach *via* an OpenMP environment (courtesy Kay Diederichs, University of Konstanz; available at http://cns-online.org). Crystallographic symmetry is then applied to the structure factors, and the target function and its derivatives with respect to structure factors are evaluated. Symmetry operators are applied to the derivatives of the target function with respect to the structure factors followed by inverse Fourier transform. Using the chain rule, derivatives of the target function with respect to atomic parameters are then computed by multiplication and summation over the local neighborhood around each atom of the derivatives of the electron density with respect to atomic parameters.

Although the original SGFFT method was developed with an isolated-atom description of electron density and isotropic ADPs, it is generalizable to aspherical Cartesian Gaussian multipoles and anisotropic ADPs. All that is needed are formulae for the electron density and the derivatives of the electron density with respect to atomic parameters, which then can be inserted into equations (29) and (40) of Brünger (1989 ▶). In the following sections, we develop these necessary formulae.

### 2.2. Isolated-atom Gaussian density

The key mathematical property of Gaussians with respect to efficient calculation of structure factors is that they are an eigenfunction of the Fourier transform (FT). In other words, a Gaussian in real space transforms to a Gaussian in reciprocal space and *vice versa*. Consider the canonical spherically symmetric Gaussian atomic scattering factor (Agarwal, 1978 ▶),
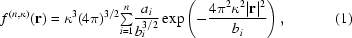
where *a*_*i*_ and *b*_*i*_ are constant parameters fitted to *ab initio* calculations on isolated atoms (this work is based on a sum of six Gaussians; *n* = 6; Su & Coppens, 1998 ▶), κ is an expansion/contraction parameter used to adjust the width of the density and **r** is a position vector relative to the center of the atom. Its FT is given by
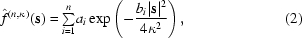
where **s** is the reciprocal-lattice vector and we have used the FT definition given in Appendix *A* [App appa]. The reciprocal-lattice vector is **s** = **h** ^*t*^ **A** ^−1^ = (**A** ^−1^)^*t*^ **h**, where **h** is a column vector with the Miller indices of a Bragg reflection and **A** is the fractional­ization matrix that transforms coordinates **r** with respect to a Cartesian basis to fractional coordinates **r**_frac_ as defined in a crystallographic basis set. The Debye–Waller factor (Waller, 1923 ▶) is given by
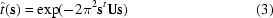
in reciprocal space, where each element of the symmetric positive-definite matrix **U** is defined *via* a Cartesian basis consistent with PDB ANISOU records (Trueblood *et al.*, 1996 ▶; Grosse-Kunstleve & Adams, 2002 ▶). Multiplication of (3) by the atomic form factor from (2) gives the scattering factor
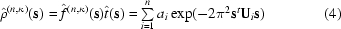
based on **U**_*i*_ that are defined by
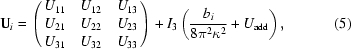
where *U*_add_ is the artificial isotropic increase or decrease in the ADP discussed above and *I*_3_ is a 3 × 3 identity matrix. Removal of *U*_add_ analytically from each structure factor after the FT is straightforward. The only difference, therefore, between each **U**_*i*_ is the isolated-atom scattering parameter *b*_*i*_.

Application of the inverse FT to (4) gives the real-space anisotropic electron density
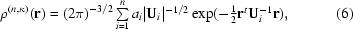
where |**U** *_i_*| is the determinant of matrix **U** *_i_* and **U** *_i_* ^−1^ is its inverse. This expression can also be viewed as the convolution of the Gaussian form factor of (1) with the inverse Fourier transform of the Debye–Waller factor of (3). Although the underlying isolated-atom scattering factor is spherically symmetric, convolution with anisotropic ADPs can lead to an angular dependence in ρ^(*n*,κ)^(**r**). Using the relationship that *B* = 8π^2^ *U*, one can show that (6) reduces to the isotropic density expression reported by Brünger in equation (16) of Brünger (1989 ▶) if all diagonal elements of **U**_*i*_ are equal to *U*_iso_ + *b_i_*/8π^2^ + *U*_add_ with zero off-diagonal components.

### 2.3. Polarizable atomic multipole electron density

For the derivation of an atomic multipole expansion from a collection of point charges we begin with the Taylor expansion of the electric potential *V*(**r**) at **r** arising from *n* partial point charges that represent the electron density of an atom,
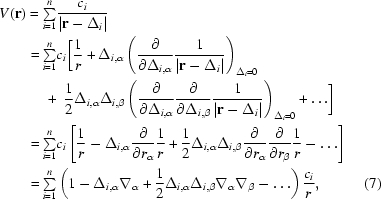
where Δ_*i*_ is the position of partial charge *c*_*i*_, ∇_α_ = ∂/∂*r*_α_ is one component of the del operator, α ∈ {*x*, *y*, *z*} and the Greek subscripts {α, β} represent the use of the Einstein summation convention for summing over tensor elements (Stone, 1996 ▶). We omit the constant factor of 1/4π∊_0_ throughout for com­pactness. Let the monopole, dipole and traceless quadrupole moments be defined as
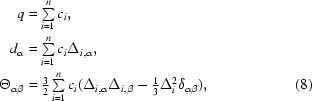
where removal of the trace in the definition of the quadrupole moment is allowed because the potential satisfies the Laplace equation (*i.e.* ∇^2^ *V* = 0). Substitution of the relationships in (8) into the final expression of (7) gives the electric potential in terms of a Cartesian multipole expansion, which we truncate at quadrupole order
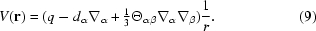
We now replace the Coulomb potential of (9) with the potential from the sum of Gaussians from (1), which is given by
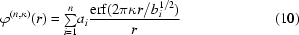
and find
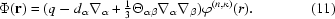
We now introduce unique superscripts on the charge, dipole and quadrupole Gaussian basis sets, denoted by {*n_q_*, *n_d_*, *n*_Θ_} and {κ*_q_*, κ*_d_*, κ_Θ_}, to allow them to differ in number and width.
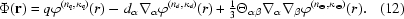
The potential of the charge density of (12) quickly approaches the Coulomb potential as *r* increases since the error function goes to unity such that at large *r* this potential satisfies the Laplace equation and the use of a traceless quadrupole tensor is still justified. Application of the Laplace operator to both sides of (12) gives the negative of a continuous charge density based on Cartesian Gaussian multipoles, 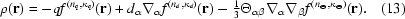
In crystallography the convention is that electron density is positive, so we will keep the negative sign. Therefore, a negative partial charge equates to positive scattering density.

Inclusion of ADPs is described by convolution of (13) with the real-space temperature factor,
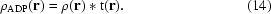
Based on the convolution differentiation rule
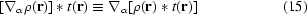
the solution to (14) is given by substituting for *f*(**r**) in (13) with the corresponding ρ(**r**) from (6) to give
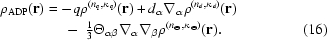
However, since *q* only represents partial atomic charges, the contributions from valence and core electrons need to be added. Additionally, the AMOEBA force field divides each atomic dipole moment into permanent (**d**) and induced (**u**) contributions to account for polarization. Therefore, we construct the total atomic electron density at a location **r** relative to the center of atom *j* by adding the contribution of core and valence electron density to (16) and splitting the dipole into permanent and induced components to give
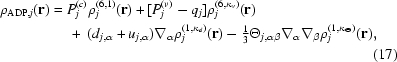
where *P_j_* ^(*c*)^ is the integer number of core electrons (carbon has two) and *P_j_* ^(*v*)^ is the integer number of valence electrons (carbon has four). The superscripts on the anisotropic Gaussian form factors *ρ_j_* ^(*n*,κ)^(**r**) have been made explicit for our model. We make the reasonable choice of using the isolated-atom scattering parameters for both core and valence electron densities. The width of the core electron density is frozen at the isolated-atom description (κ = 1) based on the observation that chemical bonding does not perturb it significantly (Hansen & Coppens, 1978 ▶). On the other hand, the width of the valence electron density expands or contracts relative to the isolated-atom model owing to a gain or reduction, respectively, of electron density from or to covalently bonded atoms. This effect is modeled by the width parameter of the valence density κ_*v*_. In this work, the dipole and quadrupole densities are described by a single Gaussian (*n*_*d*_ = *n*_Θ_ = 1) based on *a* and *b* parameters set to unity. The widths of the dipole and quadrupole densities are controlled by the κ_*d*_ and κ_Θ_ parameters. In this work, the width parameters {κ_*v*_, κ_*d*_, κ_*Θ*_} are optimized against the diffraction data for each AMOEBA multipole type. The multipole moments are fixed by the AMOEBA force field and are not refined against the data.

The partial derivatives through second order of the anisotropic and aspherical density defined in (6), which are required for the real-space multipolar density given in (17), are
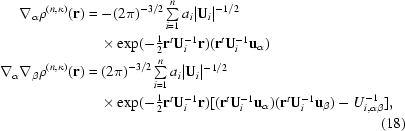
where **u**_α_ is a unit vector in the α direction with α ∈ {*x*, *y*, *z*}. In addition, the third-, fourth- and fifth-order terms of the expansion are presented as supplementary information along with a Mathematica notebook.^1^

To the best of our knowledge, (17) is the first expression reported in the literature for a real-space form factor that is the convolution of an atomic multipolar electron density with anisotropic ADPs. This equation opens the door to exploring precise polarizable atomic multipole refinements in tandem with efficient computation of structure factors *via* FFT.

**Figure 1:**
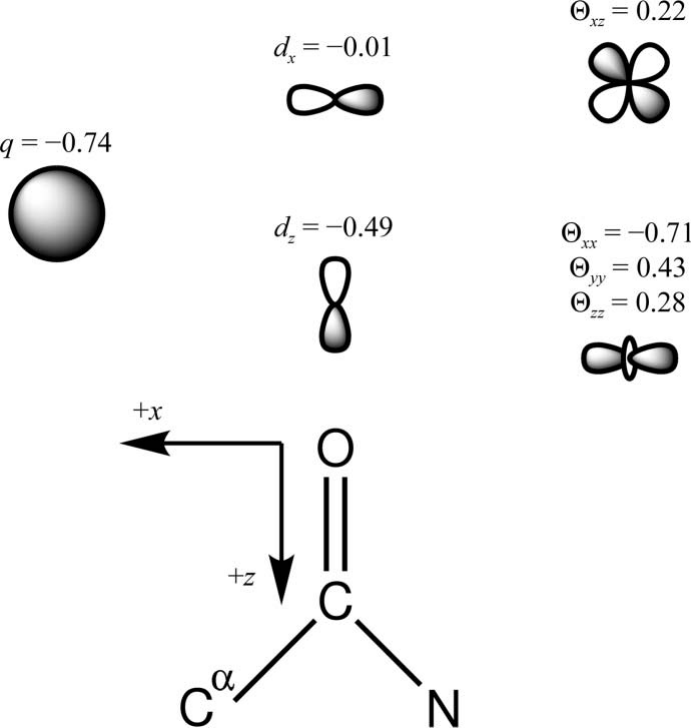
The local multipole frame of the carbonyl O atom of the peptide backbone is shown. The positive *z* axis is along the C=O bond and the *x* axis is chosen in the O=C—C^α^ plane in the direction of the C^α^ atom. The *y* axis is directed into the page in order to achieve a right-handed coordinate system. Also shown are the nonzero multipole moments of the O atom and a qualitative representation of their shape. The *d*_*z*_ Cartesian Gaussian dipole (in Debye units) places electron density along the C=O bond, while the trace of the Cartesian Gaussian quadrupole (in Buckingham units) positions electron density approximately at lone-pair positions.

Given a molecular conformation, the AMOEBA permanent multipole moments for each atom in the global coordinate frame (*q*, **d**, Θ) are converted *via* rotation from a local frame. For example, as shown in Fig. 1, ▶ the *z* axis of the local frame for the carbonyl O atom of the peptide bond is in the direction of the bond to the carbonyl C atom. Its positive *x* axis is located in the O=C—C^α^ plane in the direction of the C^α^ atom and the *y* axis is chosen to give a right-handed coordinate system (Ren & Ponder, 2002 ▶). The induced dipole (**u**) on each atom is determined *via* a self-consistent field (SCF) calculation, where the field is a sum of contributions from the permanent atomic multipoles and induced dipoles. The AMOEBA polarization model is described in greater detail in work by Ren & Ponder (2002 ▶).

### 2.4. Derivatives of the electron density

#### 2.4.1. Atomic coordinates

As a simplification, the derivation up to this point has assumed that the atomic center was the origin of the coordinate system. However, for this section on the derivatives with respect to atomic coordinates we place atom *j* at **r**_*j*_ in the global frame. In order to keep the derivation manageable, we split the total electron density into that produced by permanent charges ρ_perm_ and that of induced charges ρ_ind_,
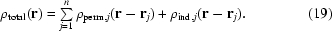
The derivative of the permanent multipole electron density of atom *j* with respect to the α coordinate of atom *j* is given by
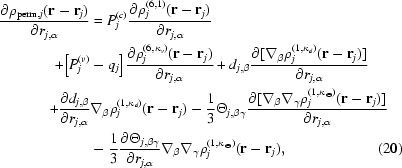
where the derivative of the dipole and quadrupole densities are each composed of two terms owing to the chain rule. As described above, the dipole and quadrupole moments of each atom are implicitly a function of its coordinates and the coordinates of a few of its bonded neighbors (atoms *k*) that define the local frame of the multipole. Therefore, the derivative of the permanent multipole electron density of atom *j* with respect to the α coordinate of atoms *k* must also be considered,
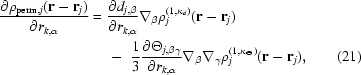
where the derivatives of spherically symmetric terms are zero with respect to the coordinates of atom *k* because they have no dependence on the orientation of the local frame. Note that the partial derivative of an anisotropic and aspherical density tensor with respect to an atomic coordinate is the negative of the partial derivatives given in (18), simply due to the negative sign on **r** *_j_*. The derivatives of the polarizable density with respect to atomic coordinates are very specific to the AMOEBA electrostatic model and are discussed in Appendix *B* [App appb]. However, we note that computing the derivatives of a polarizable density with respect to atomic coordinates is *O*(*n* ^2^log*n*) using PME, which quickly becomes the most expensive part of the overall calculation.

#### 2.4.2. ADPs

The derivative of the anisotropic electron density of atom *j* with respect to an anisotropic displacement parameter *U*_*j*,τυ_ is given by
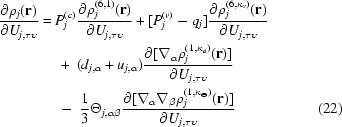
and requires the partial derivatives of the Cartesian Gaussian tensors with respect to ADP components. Introducing a few relationships facilitates their presentation. Firstly, based on the equality
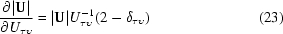
we have
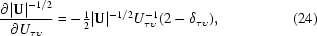
where the Kronecker delta δ_τυ_ is unity for diagonal elements of **U** and zero otherwise. Differentiating an identity from matrix algebra **U** ^−1^ **U** = **I** gives the following relationship
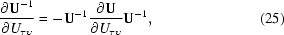
which makes it possible to differentiate **U** instead of its inverse. This is preferred since only one or two elements of ∂**U**/∂*U*_τυ_ are equal to unity and the rest are zero. Specifically, a single element is equal to unity if τ equals υ, while two elements are equal to unity otherwise, since *U*_τυ_ and *U*_υτ_ represent the same variable in this case. For convenience, we define a 3 × 3 matrix **J** ^(τυ)^,
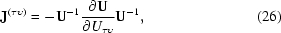
and based on the chain rule we have
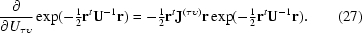
Differentiating (6) with respect to **U**_τυ_ and using (24), (27) and the product rule gives
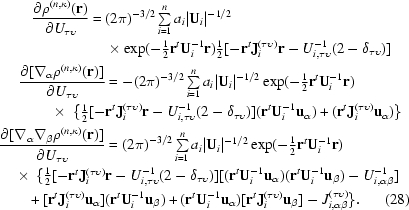

               

#### 2.4.3. Gaussian width

The Gaussian width parameter κ controls radial expansion and contraction of the Cartesian Gaussian multipoles. Analogous parameters are used to optimize the STOs within the Hansen and Coppens scattering model (Hansen & Coppens, 1978 ▶). The derivative of the electron density with respect to this parameter is similar to the gradient for the ADP parameters. Two chain-rule terms are necessary. Firstly, the gradient of the normalizing term
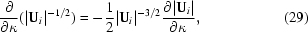
where
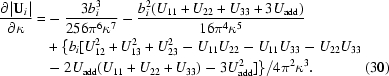
Secondly, the gradient of the inverse ADP matrix is most conveniently expressed using the gradient of the original ADP matrix,
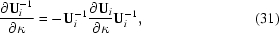
where
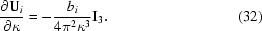
For convenience the matrix **J**_*i*_^(κ)^ is defined to more compactly represent this result, 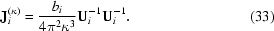
Differentiating (6) with respect to κ and using (29), (33) and the product rule gives
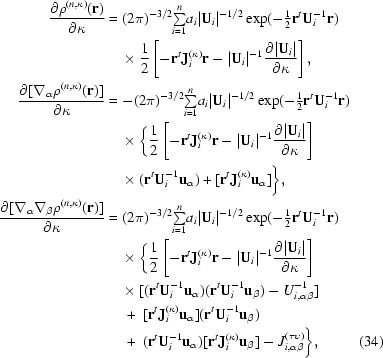
together with the third- and fourth-order terms available as supplementary information^1^.

### 2.5. Fourier transform of the polarizable atomic multipole electron density

Remarkably, the FT of the anisotropic and aspherical density given in (17) is simply
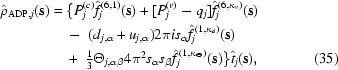
where the dipole and quadrupole terms in (35) depend on the FT of the partial derivatives defined in (18). Through fifth order the reciprocal-space tensors are
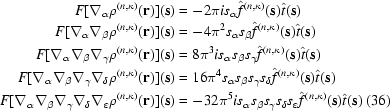
and in compressed tensor notation the general expression for order *u* + *v* + *w* is
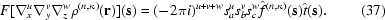
This expression is considerably more compact than any reported previously for an aspherical scattering factor in reciprocal space, particularly the formulation based on STOs and spherical harmonics (Hansen & Coppens, 1978 ▶). Notably, our formulation has no dependence on cumbersome Fourier Bessel transforms of Slater-type functions (Dawson, 1967*a* ▶; Hansen & Coppens, 1978 ▶; Su & Coppens, 1990 ▶). Our equation (35) has been implemented by ‘direct summation’ for com­parison to the performance of the FFT algorithm.

## 3. Scattering models

Four scattering models were implemented by modifying and combining the *CNS* (Brünger *et al.*, 1998 ▶) and *TINKER* (Ponder, 2004 ▶) code bases. The scattering models were added to the *CNS* code base, while *TINKER* was used to compute AMOEBA chemical forces and to supply *CNS* with polarizable multipoles in the global frame.

### 3.1. Isolated atom

The first scattering model (‘IAM’) is the conventional IAM based on the relativistic elastic scattering factors described by Su & Coppens (1998 ▶).

### 3.2. Isolated atom with inter-atomic scattering

The second scattering model (‘IAM–IAS’) augments the IAM with inter-atomic scattering sites at bond centers (Afonine *et al.*, 2007 ▶). Unlike the model of Afonine and coworkers, our implementation does not include IAS sites at lone pairs or at the center of aromatic rings. We have neglected these sites based on the rationale that the AMOEBA electrostatic model is sufficient to capture these details of the electron density, which we provide further evidence for below when discussing the refinement of a Tyr-Gly-Gly tripeptide.

In our approach, chemically equivalent bonds are constrained to use the same IAS parameters. Charge density that is added to or removed from bond centers is exactly balanced by changing the net charge of the bond-defining atoms. For example, a bond charge of −0.2 e requires atomic charge increments that sum to 0.2 e. In this way, all molecules retain their original net charge. Each bond type requires three parameters: the charge increments of both atoms and the Gaussian width of the scattering site. Bond types are defined based on the concatenation of the AMOEBA force-field atom types.

### 3.3. AMOEBA

The third scattering model (‘AMOEBA’) is based on the polarizable atomic multipoles of the AMOEBA force field. Each chemically unique multipole type requires three Gaussian width parameters as described in §[Sec sec2]2. The induced dipoles were iterated to self-consistency using PME whenever any atomic coordinates were changed during refinement (Darden *et al.*, 1993 ▶; Sagui *et al.*, 2004 ▶; Essmann *et al.*, 1995 ▶).

### 3.4. AMOEBA with inter-atomic scattering

The final scattering model (‘AMOEBA–IAS’) augments AMOEBA electrostatics with inter-atomic scattering sites. It became clear during the course of this study that an atomic multipole expansion truncated at quadrupole order is insufficient to capture bond charge density for most molecular geometries. This is consistent with theoretical observations by Stone and coworkers that the convergence of a distributed multipole analysis (DMA) may be improved by using both atoms and bond centers as expansion sites (Stone & Alderton, 1985 ▶; Stone, 2005 ▶). Furthermore, experimental data from the X-ray scattering of diamond and silicon, simple examples of tetrahedral bonding geometry, are explained by the superposition of one atomic octopole moment and one atomic hexadecapole moment (Dawson, 1967*a* ▶,*b* ▶). The characteristics of the four scattering models are further clarified below with respect to four peptide test cases.

The following computational details were constant across all of the refinements. The isotropic ADP offset *U*_add_ was set to 1/(4π^2^), which is equivalent to *B*_add_ = 8π^2^ *U*_add_ = 2, the FFT grid factor to 0.33 (as appropriate for crystal structures at sub­atomic resolution), and the electron-density cutoff around each atom was 18 (specified by the *E*_lim_ parameter in *CNS*). These conservative parameters led to close agreement between direct summation and FFT computation of structure factors. The *CNS* parameter *w*_A_ that controls the weighting of X-ray target function relative to the force-field energy was set to 1.0, although we also tested 0.2.
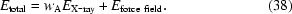
This raised *R*_free_ values by less than 0.1% and lowered the AMOEBA potential energy differences between refinements presented below, but did not alter any trends or our conclusions. It should be noted that force-field restraints are not necessarily required for refinement at subatomic high resolution. However, their use in this study gives an insight into the relative energetic cost of the structural changes arising from differences in the four scattering models. A modified version of the refine.inp *CNS* task file was used for all refinements using the MLI target function.

## 4. Applications

To demonstrate the behavior of X-ray refinements based on Cartesian Gaussian multipoles, we present two sets of applications. The first set is simply to illustrate the performance of direct summation *versus* FFT and SGFFT computation of structure factors as a function of system size. The second set describes refinements on a series of four peptide crystals that diffract to 0.59 Å resolution or better. All examples use the AMOEBA force field for chemical forces, instead of the default *CNS* force field based on Engh & Huber parameters (Engh & Huber, 1991 ▶). Although the refinements were performed in the native space group of each crystal, AMOEBA energies and gradients as computed using the *TINKER* code base required expanding to *P*1. This did not increase the number of refined variables, but suggests the need for an AMOEBA code that takes advantage of crystal symmetry.

### 4.1. Runtime scaling on protein data sets

**Figure 2:**
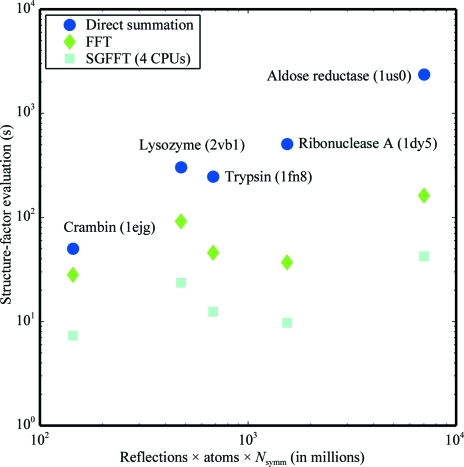
The scaling of the Cartesian Gaussian multipole model, truncated at quadrupole order, is plotted on a log–log scale for computation of the intensity-based maximum-likelihood target function (MLI) for direct summation, FFT and SGFFT. Direct summation scales linearly with the product of the number of atoms, the number of reflections and the number of symmetry operators. Computation of the crystallographic target function by FFT of the Cartesian Gaussian multipole electron density shows a speedup of a factor of between 1.8 and 14.5 compared with direct summation. A further speedup factor of nearly four is achieved using the SGFFT method on a four-processor machine.

Evaluation of the target function and its derivatives by direct-summation calculation of structure factors *via* (35) and (36) is *O*(*N*_atoms_ × *N*_reflections_ × *N*_symm_). Alternatively, the FFT algorithm based on (17) and (18) is *O*(*N*_grid_ × log*N*_grid_), where the number of grid points *N*_grid_ depends on the resolution of the diffraction data. Aspherical refinements based on the Hansen–Coppens formalism are currently limited to direct summation, since the real-space form of the electron density convolved with ADPs is unknown. Therefore, the performance of X-ray refinements based on Cartesian Gaussian multipoles and FFT is of particular interest. The results are summarized in Table 1 ▶ and are plotted in Fig. 2 ▶. Although the performance difference is only about a factor of two for the small protein crambin, over an order of magnitude improvement is achieved for both ribonuclease A and aldose reductase. Parallelization with the SGFFT method results in a further significant speedup (a speedup of a factor of nearly four relative to a single processor on a four-processor machine).

### 4.2. Refinement of peptide crystals

In principle, a more precise scattering model based on Cartesian Gaussian multipoles with coefficients from the AMOEBA electrostatics model should improve the quality of refinements relative to the IAM as judged by both *R*_free_ and the potential energy of the asymmetric unit. Furthermore, the quality of the AMOEBA potential energy function can also be assayed, since it is reasonable to expect that potential energy and *R*_free_ should be correlated.

The peptide crystals studied include YG_2_ (Pichon-Pesme *et al.*, 2000 ▶), cyclic P_2_A_4_ (Dittrich *et al.*, 2002 ▶) and AYA with three waters or with an ethanol molecule (Chęcińska, Forster *et al.*, 2006 ▶; Chęcińska, Mebs *et al.*, 2006 ▶). Detailed descriptions of the unit-cell parameters, number of atoms, resolution and measured reflections are given in Table 2 ▶. The refinement results are summarized in Table 3 ▶ and compared with previous work below.

#### 4.2.1. YG_2_

The *R*_free_ values of the IAM and IAM–IAS refinements of YG_2_ (4.60 and 3.86%, respectively) are slightly lower than those reported by Afonine and coworkers (4.72 and 4.06%, respectively; Afonine *et al.*, 2007 ▶). The *R*_free_ value of the AMOEBA–IAS refinement (3.50%) is a significant improvement. The *R*_work_ value (3.17%) of the AMOEBA–IAS refinement is also lower and is comparable to multipolar refinements reported by Volkov and coworkers using transferred or refined multipole coefficients (3.66% and 3.42%, respectively; Volkov *et al.*, 2007 ▶). Cross-validation-based comparisons are unavailable in this case. We note that the AMOEBA–IAS refinement used a reflections-to-parameters ratio of 11.1, which is slightly higher than the value of 10.6 reported by Volkov and coworkers using refined multipole coefficients. This is computed based on the number of reflections reported in Table 2 ▶ and the number of parameters given in Table 3 ▶.

**Figure 3:**
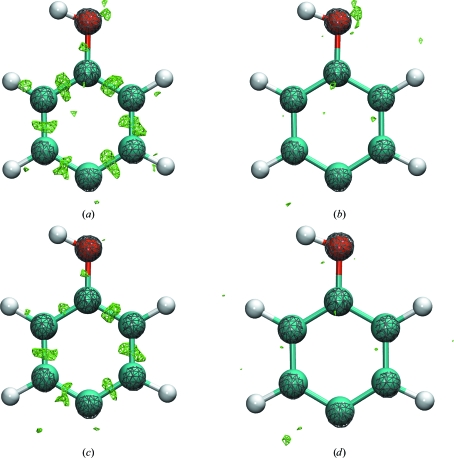
(*a*) IAM, (*b*) IAM–IAS, (*c*) AMOEBA and (*d*) AMOEBA–IAM refinements, respectively, for GY_2_. The *F*_o_ − *F*_c_ and 2*F*_o_ − *F*_c_ σ_A_-weighted electron-density maps are contoured at 3.5σ and shown in green and gray, respectively. Both the IAM and AMOEBA models fail to explain the electron density at bond centers seen in the data. In addition, the IAM model does not account for lone-pair density on the O atom.

Electron-density maps of the tyrosine ring for the four scattering models are shown in Fig. 3 ▶, which lend visual insight into their properties. The non-H atom positions are apparent in the 2*F*_o_ − *F*_c_ contours for each refinement. The standard IAM scattering model underestimates the electron density at bond centers and at the oxygen lone-pair sites, as shown by the *F*_o_ − *F*_c_ con­tours. Our IAM–IAS scattering model explains the electron density at bond centers, but does not capture lone-pair electron density. Conversely, the AMOEBA model places electron density approximately at the lone-pair positions but not at bond centers. Finally, the AMOEBA–IAS model explains much of the lone-pair and bonding electron densities.

#### 4.2.2. P_2_A_4_

The *R*_free_ values of our IAM and IAM–IAS refinements of P_2_A_4_ (3.73 and 3.01%, respectively) agree closely with the values of Afonine and coworkers (3.63 and 3.23%, respectively; Afonine *et al.*, 2007 ▶). The *R*_free_ value of the AMOEBA–IAS refinement (2.94%) is lower by 0.07%, which is the least amount of improvement seen for AMOEBA–IAS relative to IAM–IAS in this study. The *R*_work_ value (2.86%) of the AMOEBA–IAS refinement is slightly higher, but com­parable to those reported by Volkov and coworkers using transferred or refined multipole coefficients (2.60% and 2.53%), although this work uses a higher reflections-to-parameters ratio (50.3 compared with 43.6; Volkov *et al.*, 2007 ▶). As for YG_2_, cross-validation was not performed. The similarity of the *R* values for YG_2_ and P_2_A_4_ between the AMOEBA–IAS refinements and the multipolar refinements of Volkov and coworkers is consistent with the principle that bond scattering sites capture density that is represented by higher order atomic moments missing in the AMOEBA model (octopole and hexadecapole).

**Figure 4:**
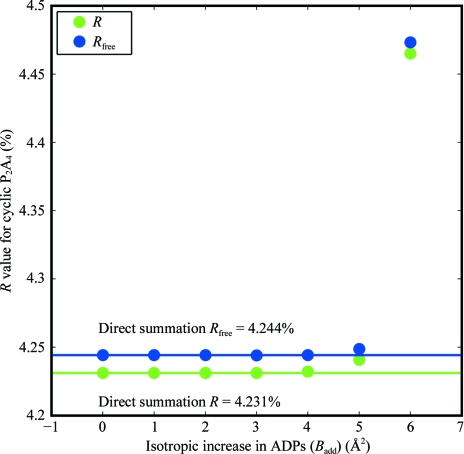
The precision of numerical computation of the *R*_work_ and *R*_free_ values *via* FFT is compared with analytic direct summation as a function of the isotropic increase *B*_add_ in ADP parameters for P_2_A_4_ under the AMOEBA scattering model. Note that *B*_add_ = 8π^2^ *U*_add_.

In Fig. 4 ▶ the precision of the *R*_work_ and *R*_free_ values computed using discrete FTs are compared with analytic direct summation for P_2_A_4_ under the AMOEBA scattering model. Agreement to four decimal places is seen for *B*_add_ values between 0 and 3 Å^2^, which serves as validation of the correctness of (17) and (35). These results support the conclusion that FFT-based computation of structure factors is appropriate for anisotropic and aspherical scattering models.

#### 4.2.3. AYA

The AYA data sets were chosen because of the extremely low temperature achieved during the measurement of structure factors (9 K for AYA + three waters and 20 K for AYA + ethanol). For AYA + water, Chęcińska and coworkers (Chęcińska, Forster *et al.*, 2006 ▶; Chęcińska, Mebs *et al.*, 2006 ▶) originally reported an *R* value of 2.4%, which is in agreement with the *R* value of our IAM refinement (2.67%). Addition of IAS lowered the *R*_free_ statistic from 2.71% to 2.39%, while addition of polarizable atomic multipole electron density showed a further improvement to an *R*_free_ of 1.95%. For AYA + ethanol the *R*_work_ value of the IAM (3.20%) is comparable to that reported originally by Chęcińska and coworkers (2.9%). IAM–IAS lowered *R*_free_ from 3.33 to 2.49%, while AMOEBA–IAS achieved 2.08%.

### 4.3. Refinement summary

**Figure 5:**
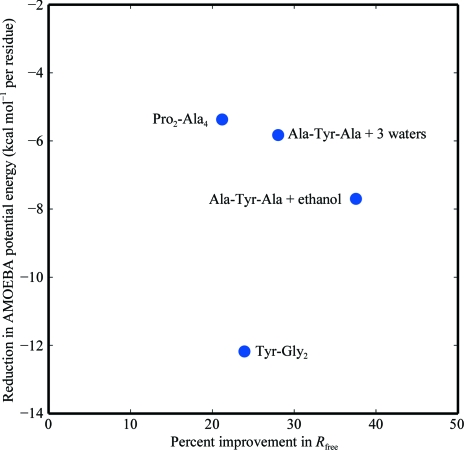
The improvement arising from the AMOEBA–IAS scattering model, relative to the IAM model, is plotted as a function of relative percentage improvement in *R*_free_ value and the relative AMOEBA potential energy per residue. For all data sets, the best *R*_free_ value and lowest potential energy per residue were achieved using the AMOEBA–IAS scattering model. 1 kcal mol^−1^ = 4.186 kJ mol^−1^.

The results for all four peptide refinements are summarized in Fig. 5 ▶. In every case, use of the AMOEBA–IAS scattering model relative to the IAM scattering model lowered both *R*_free_ and the potential energy of the crystal. When the IAM scattering model is used, molecular conformations are highly strained to compensate. For example, H—C atom bonds are too short because the IAM model centers electron density at the hydrogen nucleus. In the crystal structures, this electron density is shifted towards the C atom. As the description of the electron density is improved, the molecular conformation relaxes by approximately 16 kJ mol^−1^ per residue. The precise amount of relaxation depends on the weighting between the crystallographic target and the force field. Unrestrained refinements with an IAM scattering model could adopt even more unphysical conformations. This suggests that accurate chemical restraints are necessary even for ultrahigh-resolution refinements unless an anisotropic and aspherical scattering model is used.

**Figure 6:**
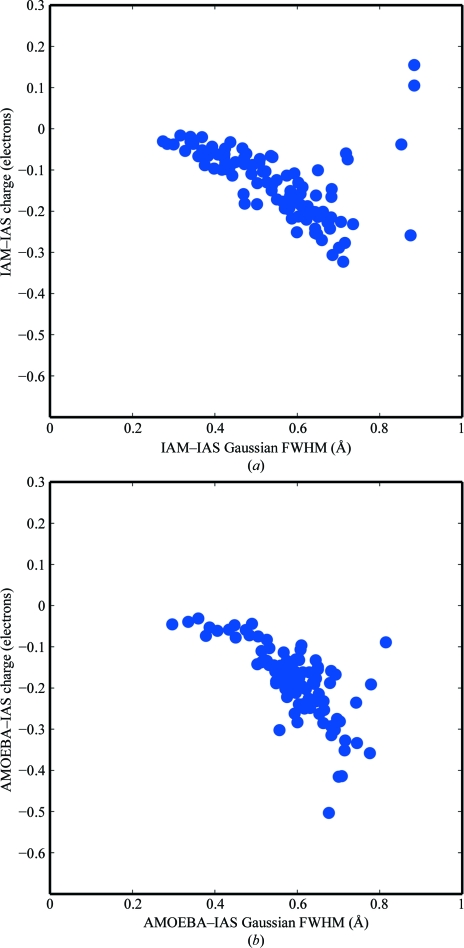
For the inter-atomic scattering sites of the IAM–IAS (*a*) and AMOEBA–IAS (*b*) scattering models, the refined Gaussian full-width at half-maximum (FWHM) is plotted *versus* partial charge magnitude. The majority of charges for the IAM–IAS model and all charges for the AMOEBA–IAS are negative. The sub-angstrom FWHM values are consistent with very localized bond densities.

In Fig. 6 ▶, we present plots of the IAS sites that were refined for each peptide system. Their Gaussian full-width at half-maximum (FWHM) is plotted against charge magnitude for both the IAM–IAS and the AMOEBA–IAS models. The majority of the charges under the IAM–IAS model and all of the charges under the AMOEBA–IAS model refined to negative partial charge values (or positive scattering density), which is consistent with the physical concentration of charge density at chemical bonds. The similarity of the refined charges between the IAM–IAS and the AMOEBA–IAS models suggests that an atomic multipole description of electron density truncated at quadrupole order underestimates density at trigonal and tetrahedral bond centers.

## 5. Conclusions

Cartesian Gaussian multipoles offer an efficient alternative to the Hansen and Coppens formulation of aspherical scattering. They eliminate the use of Slater-type functions and allow structure factors to be computed by FFT. Numerical tests show that that FFT and direct-summation implementations of Cartesian Gaussian multipoles agree to high precision. For subatomic resolution biomolecular data sets such as ribo­nuclease A and aldose reductase, parallelized computation of structure factors using the SGFFT method results in a speedup of one to two orders of magnitude compared with direct summation.

Ideally, a force-field electrostatics model should be accurate enough to explain the electron density observed in X-ray diffraction experiments. Although the AMOEBA polarizable multipole force field energetic model shows promise, truncation of the permanent moments at quadrupole order systematically underestimates electron density at bond centers. Our results suggest that the added computational expense of including hexadecapole moments in the atomic scattering factor computation is justified. As supplementary information we have provided a Mathematica notebook and formulae that allow computation of Cartesian Gaussian multipoles up to the fourth order in anticipation of further improvements to force fields.

In the near future, we will present the results of applying our polarizable atomic multipole refinement methodology to macromolecules. For ultrahigh-resolution macromolecular data sets, such as HEWL at 0.65 Å (Wang *et al.*, 2007 ▶), our scattering model significantly improves refinement statistics, as it does for the simpler peptide cases presented here. Equally exciting will be the use of the AMOEBA force field and in particular the electrostatic forces to orient water molecules in the absence of clear H-atom electron density. We anticipate that refinement of hydrogen-bonding networks will enhance the usefulness of X-ray crystallography experiments with respect to explaining p*K*_a_ shifts, ligand-binding affinities and enzymatic mechanisms.

## Supplementary Material

Supplementary material file. DOI: 10.1107/S0907444909022707/dz5164sup1.pdf

Supplementary material file. DOI: 10.1107/S0907444909022707/dz5164sup2.pdf

## Figures and Tables

**Table 1:** Comparison of computational efficiency of direct-summation, FFT and SGFFT methods for the computation of the Cartesian Gaussian multipole scattering factors The permanent multipole expansion was truncated at atomic quadrupoles and polarization was included *via* induced dipoles. The FFT method shows a speedup factor of 1.8–14.5 relative to direct summation. Parallelization by SGFFT provided an additional factor of 3.7–3.9 using four processors. All calculations were performed on a MacPro workstation with 2 × 2.66 GHz Dual Core Intel Xeon processors.

PDB code	Atoms	Reflections	*N*_symm_	Atoms × reflections × *N*_symm_ × 10^−6^	Direct (s)	FFT (s)	Direct/FFT	SGFFT (s)	Direct/SGFFT
1ejg	642	112209	2	144.1	49.9	28.1	1.8	7.3	6.8
2vb1	2544	187165	1	476.1	301.8	91.5	3.3	23.6	12.8
1fn8	4294	158550	1	680.8	245.1	45.8	5.4	12.4	19.8
1dy5	4835	159422	2	1541.6	505.6	37.0	13.7	9.7	52.1
1us0	6865	511265	2	7019.7	2346.2	162.3	14.5	42.3	55.5

**Table 2:** Refinement systems

Molecule	Space group and unit-cell parameters (Å, °)	Non-H atoms	H atoms	Bonds	*d*_min_ (Å)	Reflections
YG_2_	*P*2_1_2_1_2_1_, *a* = 7.98, *b* = 9.54, *c* = 18.32	22	19	40	0.43	4766
P_2_A_4_	*P*2_1_2_1_2_1_, *a* = 10.13, *b* = 12.50, *c* = 19.50	35	36	72	0.37	24878
AYA + 3 waters	*P*2_1_, *a* = 8.12, *b* = 9.30, *c* = 12.53, β = 91.21	26	27	50	0.59	5019
AYA + ethanol	*P*2_1_, *a* = 8.85, *b* = 9.06, *c* = 12.36, β = 94.56	26	27	52	0.59	5258

**Table 3:** Refinement statistics and the relative AMOEBA potential energy per asymmetric unit are given for four small peptide crystals using the IAM, IAM–IAS, AMOEBA and AMOEBA–IAS scattering models In all cases, the lowest *R*_free_ was found using the AMOEBA–IAS scattering model. Furthermore, the structure with the lowest AMOEBA potential energy per asymmetric unit also corresponded to AMOEBA–IAS refinement.

				*R*_work_/*R*_free_ (%)		
Molecule	Scattering model	*N*_param_	*N*_data_/*N*_param_	*I*_obs_/σ(*I*_obs_) > 0	*I*_obs_/σ(*I*_obs_) > 3	Energy† (kcal mol^−1^)
YGG	IAM	274	17.4	4.73/4.74	4.41/4.60	36.5
	IAM–IAS	349	13.7	3.93/4.01	3.59/3.86	7.2
	AMOEBA	355	13.4	4.50/4.56	4.16/4.37	6.8
	AMOEBA–IAS	430	11.1	3.54/3.72	3.17/3.50	0.0
PPAAAA	IAM	339	73.4	4.25/4.22	3.65/3.73	32.2
	IAM–IAS	417	59.7	3.56/3.48	3.00/3.01	18.3
	AMOEBA	417	59.7	4.24/4.23	3.69/3.77	12.9
	AMOEBA–IAS	495	50.3	3.42/3.42	2.86/2.94	0.0
AYA + 3 waters	IAM	342	14.7	2.75/2.79	2.67/2.71	17.5
	IAM–IAS	411	12.2	2.24/2.47	2.16/2.39	4.1
	AMOEBA	423	11.9	2.40/2.55	2.31/2.47	4.7
	AMOEBA–IAS	492	10.2	1.72/2.03	1.64/1.95	0.0
AYA + ethanol	IAM	342	15.4	3.30/3.50	3.20/3.33	23.1
	IAM–IAS	423	12.4	2.32/2.66	2.21/2.49	14.8
	AMOEBA	435	12.1	3.42/3.75	3.32/3.58	3.7
	AMOEBA–IAS	516	10.2	1.90/2.25	1.79/2.08	0.0

† 1 kcal mol^−1^ = 4.186 kJ mol^−1^.

## Appendix A Fourier transform definition

The definition and notation for the Fourier transform as used in this work is given by

and the corresponding inverse Fourier transform by

## Appendix B Derivative of the polarizable electron density with respect to atomic coordinates

The total polarizable electron density arising from the induced dipole of all atoms is given by

The gradient of this density with respect to the α component of atom *j* is

The second term is nonzero only for *i* = *j* and is simple to calculate. The first term, however, depends on ∂*u*_*i*,β_/∂*r*_*j*,α_ which is the derivative of a component of the induced dipole of atom *i* with respect to the α component of atom *j*. In other words, perturbing the position of atom *j* affects not only its own scattering but that of all polarizable atoms. The induced dipole on atom *i* arises from the self-consistent crystal field multiplied by the polarizability,

where α_*i*_ is the atomic polarizability of atom *i*, **T**_*ik*_^(1)^ is a matrix of tensors that produces the field at site *i*

owing to the multipole **M**_*k*_ at site *k*

and **T**_*ik*_^(11)^ is the matrix of tensors that produces the field at site *i*

owing to the induced dipole ***u***_*k*_ at site *k*. For simplicity, we have not formulated (43) using PME electrostatics. Therefore, the sum over *k* includes all atoms in the crystal except atom *i*. The derivative of (43) with respect to coordinate *r*_*j*,α_ is given by

The first three terms on the right-hand side are not difficult to compute. However, the fourth term shows that the gradients of the polarizable scattering are *O*(*n* ^3^) without use of PME. Specifically, there are 3*n* × 3*n* induced dipole density derivatives, each of which is the sum of 3*n* terms. In this work, we have computed these derivatives by finite differences using PME, which is *O*(*n* ^2^log*n*).

## Footnotes

1 Supplementary material has been deposited in the IUCr electronic archive (Reference: DZ5164). Services for accessing this material are described at the back of the journal.
