# Supplementary material for: Polarizable atomic multipole X-ray refinement: application to peptide crystals
Source: Acta Crystallogr D Biol Crystallogr. 2009 Aug 14;65(Pt 9):952–65. doi: 10.1107/S0907444909022707 (PMC2733883; doi:10.1107/S0907444909022707)
Supplement: Supplementary file 1 [file d-65-00952-sup1.pdf]

Supplementary Information: Higher Order Cartesian Gaussian Multipole Tensors

Although the AMOEBA force field does not include octopole or hexadecapole moments, future scattering models based on Cartesian Gaussian multipoles may. Therefore, we present the 3rd, 4th and 5th order tensors and the derivatives of the 3rd and 4th order tensors with respect to both ADP and κ parameters for future convenience.

Cartesian Gaussian Tensors of 3rd, 4th and 5th Order

$$\nabla_\alpha\nabla_\beta\nabla_\gamma\rho(\mathbf{r}) = -(2\pi)^{-3/2}\sum_{i=1}^{n} a_i\left|\mathbf{U}_i\right|^{-1/2} e^{-\frac{1}{2}\mathbf{r}^t\mathbf{U}_i^{-1}\mathbf{r}}
\times\begin{bmatrix}\left(\mathbf{r}^t\mathbf{U}_i^{-1}\mathbf{u}_\alpha\right)\left(\mathbf{r}^t\mathbf{U}_i^{-1}\mathbf{u}_\beta\right)\left(\mathbf{r}^t\mathbf{U}_i^{-1}\mathbf{u}_\gamma\right)-\left(\mathbf{r}^t\mathbf{U}_i^{-1}\mathbf{u}_\alpha\right)U^{-1}_{i,\beta\gamma}\\
-\left(\mathbf{r}^t\mathbf{U}_i^{-1}\mathbf{u}_\beta\right)U^{-1}_{i,\alpha\gamma}-\left(\mathbf{r}^t\mathbf{U}_i^{-1}\mathbf{u}_\gamma\right)U^{-1}_{i,\alpha\beta}\end{bmatrix}$$

$$\nabla_\alpha\nabla_\beta\nabla_\gamma\nabla_\delta\rho(\mathbf{r}) = (2\pi)^{-3/2}\sum_{i=1}^{n} a_i\left|\mathbf{U}_i\right|^{-1/2} e^{-\frac{1}{2}\mathbf{r}^t\mathbf{U}_i^{-1}\mathbf{r}}
\times\begin{bmatrix}\left(\mathbf{r}^t\mathbf{U}_i^{-1}\mathbf{u}_\alpha\right)\left(\mathbf{r}^t\mathbf{U}_i^{-1}\mathbf{u}_\beta\right)\left(\mathbf{r}^t\mathbf{U}_i^{-1}\mathbf{u}_\gamma\right)\left(\mathbf{r}^t\mathbf{U}_i^{-1}\mathbf{u}_\delta\right)\\
-\left(\mathbf{r}^t\mathbf{U}_i^{-1}\mathbf{u}_\alpha\right)\left(\mathbf{r}^t\mathbf{U}_i^{-1}\mathbf{u}_\beta\right)U^{-1}_{i,\gamma\delta}-\left(\mathbf{r}^t\mathbf{U}_i^{-1}\mathbf{u}_\alpha\right)\left(\mathbf{r}^t\mathbf{U}_i^{-1}\mathbf{u}_\gamma\right)U^{-1}_{i,\beta\delta}\\
-\left(\mathbf{r}^t\mathbf{U}_i^{-1}\mathbf{u}_\alpha\right)\left(\mathbf{r}^t\mathbf{U}_i^{-1}\mathbf{u}_\delta\right)U^{-1}_{i,\beta\gamma}-\left(\mathbf{r}^t\mathbf{U}_i^{-1}\mathbf{u}_\beta\right)\left(\mathbf{r}^t\mathbf{U}_i^{-1}\mathbf{u}_\gamma\right)U^{-1}_{i,\alpha\delta}\\
-\left(\mathbf{r}^t\mathbf{U}_i^{-1}\mathbf{u}_\beta\right)\left(\mathbf{r}^t\mathbf{U}_i^{-1}\mathbf{u}_\delta\right)U^{-1}_{i,\alpha\gamma}-\left(\mathbf{r}^t\mathbf{U}_i^{-1}\mathbf{u}_\gamma\right)\left(\mathbf{r}^t\mathbf{U}_i^{-1}\mathbf{u}_\delta\right)U^{-1}_{i,\alpha\beta}\\
+U^{-1}_{i,\alpha\beta}U^{-1}_{i,\gamma\delta}+U^{-1}_{i,\alpha\gamma}U^{-1}_{i,\beta\delta}+U^{-1}_{i,\alpha\delta}U^{-1}_{i,\beta\gamma}\end{bmatrix}$$

$$\nabla_\alpha\nabla_\beta\nabla_\gamma\nabla_\delta\nabla_\varepsilon\rho(\mathbf{r}) = -(2\pi)^{-3/2}\sum_{i=1}^{n} a_i\left|\mathbf{U}_i\right|^{-1/2} e^{-\frac{1}{2}\mathbf{r}^t\mathbf{U}_i^{-1}\mathbf{r}}
\times\begin{Bmatrix}\left(\mathbf{r}^t\mathbf{U}_i^{-1}\mathbf{u}_\varepsilon\right)\Big[\left(\mathbf{r}^t\mathbf{U}_i^{-1}\mathbf{u}_\alpha\right)\left(\mathbf{r}^t\mathbf{U}_i^{-1}\mathbf{u}_\beta\right)\left(\mathbf{r}^t\mathbf{U}_i^{-1}\mathbf{u}_\gamma\right)\left(\mathbf{r}^t\mathbf{U}_i^{-1}\mathbf{u}_\delta\right)\\
-\left(\mathbf{r}^t\mathbf{U}_i^{-1}\mathbf{u}_\alpha\right)\left(\mathbf{r}^t\mathbf{U}_i^{-1}\mathbf{u}_\beta\right)U^{-1}_{i,\gamma\delta}-\left(\mathbf{r}^t\mathbf{U}_i^{-1}\mathbf{u}_\alpha\right)\left(\mathbf{r}^t\mathbf{U}_i^{-1}\mathbf{u}_\gamma\right)U^{-1}_{i,\beta\delta}\\
-\left(\mathbf{r}^t\mathbf{U}_i^{-1}\mathbf{u}_\alpha\right)\left(\mathbf{r}^t\mathbf{U}_i^{-1}\mathbf{u}_\delta\right)U^{-1}_{i,\beta\gamma}-\left(\mathbf{r}^t\mathbf{U}_i^{-1}\mathbf{u}_\beta\right)\left(\mathbf{r}^t\mathbf{U}_i^{-1}\mathbf{u}_\gamma\right)U^{-1}_{i,\alpha\delta}\\
-\left(\mathbf{r}^t\mathbf{U}_i^{-1}\mathbf{u}_\beta\right)\left(\mathbf{r}^t\mathbf{U}_i^{-1}\mathbf{u}_\delta\right)U^{-1}_{i,\alpha\gamma}-\left(\mathbf{r}^t\mathbf{U}_i^{-1}\mathbf{u}_\gamma\right)\left(\mathbf{r}^t\mathbf{U}_i^{-1}\mathbf{u}_\delta\right)U^{-1}_{i,\alpha\beta}\\
+U^{-1}_{i,\alpha\beta}U^{-1}_{i,\gamma\delta}+U^{-1}_{i,\alpha\gamma}U^{-1}_{i,\beta\delta}+U^{-1}_{i,\alpha\delta}U^{-1}_{i,\beta\gamma}\Big]\\
-\left(\mathbf{r}^t\mathbf{U}_i^{-1}\mathbf{u}_\alpha\right)\left(\mathbf{r}^t\mathbf{U}_i^{-1}\mathbf{u}_\beta\right)\left(\mathbf{u}_\varepsilon^t\mathbf{U}_i^{-1}\mathbf{u}_\gamma\right)U^{-1}_{i,\gamma\varepsilon}\\
-\left(\mathbf{r}^t\mathbf{U}_i^{-1}\mathbf{u}_\alpha\right)\left(\mathbf{r}^t\mathbf{U}_i^{-1}\mathbf{u}_\beta\right)\left(\mathbf{r}^t\mathbf{U}_i^{-1}\mathbf{u}_\gamma\right)U^{-1}_{i,\delta\varepsilon}\\
-\left(\mathbf{r}^t\mathbf{U}_i^{-1}\mathbf{u}_\alpha\right)\left(\mathbf{r}^t\mathbf{U}_i^{-1}\mathbf{u}_\gamma\right)\left(\mathbf{r}^t\mathbf{U}_i^{-1}\mathbf{u}_\delta\right)U^{-1}_{i,\beta\varepsilon}\\
-\left(\mathbf{r}^t\mathbf{U}_i^{-1}\mathbf{u}_\beta\right)\left(\mathbf{r}^t\mathbf{U}_i^{-1}\mathbf{u}_\gamma\right)\left(\mathbf{r}^t\mathbf{U}_i^{-1}\mathbf{u}_\delta\right)U^{-1}_{i,\alpha\varepsilon}\\
+\left(\mathbf{r}^t\mathbf{U}_i^{-1}\mathbf{u}_\alpha\right)\left[U^{-1}_{i,\beta\gamma}U^{-1}_{i,\delta\varepsilon}+U^{-1}_{i,\beta\delta}U^{-1}_{i,\gamma\varepsilon}+U^{-1}_{i,\beta\varepsilon}U^{-1}_{i,\gamma\delta}\right]\\
+\left(\mathbf{r}^t\mathbf{U}_i^{-1}\mathbf{u}_\beta\right)\left[U^{-1}_{i,\alpha\gamma}U^{-1}_{i,\delta\varepsilon}+U^{-1}_{i,\alpha\delta}U^{-1}_{i,\gamma\varepsilon}+U^{-1}_{i,\alpha\varepsilon}U^{-1}_{i,\gamma\delta}\right]\\
+\left(\mathbf{r}^t\mathbf{U}_i^{-1}\mathbf{u}_\gamma\right)\left[U^{-1}_{i,\alpha\beta}U^{-1}_{i,\delta\varepsilon}+U^{-1}_{i,\alpha\delta}U^{-1}_{i,\beta\varepsilon}+U^{-1}_{i,\alpha\varepsilon}U^{-1}_{i,\beta\delta}\right]\\
+\left(\mathbf{r}^t\mathbf{U}_i^{-1}\mathbf{u}_\delta\right)\left[U^{-1}_{i,\alpha\beta}U^{-1}_{i,\varepsilon\gamma}+U^{-1}_{i,\alpha\gamma}U^{-1}_{i,\beta\varepsilon}+U^{-1}_{i,\alpha\varepsilon}U^{-1}_{i,\beta\gamma}\right]\end{Bmatrix}\qquad(1)$$

Derivative with respect to ADPs of 3rd and 4th Order Cartesian Gaussian Tensors

$$
\frac{\partial\left[\nabla_\alpha\nabla_\beta\nabla_\gamma\rho(\mathbf{r})\right]}{\partial U_{\tau\upsilon}} = -(2\pi)^{-3/2}\sum_{i=1}^{n} a_i\left|\mathbf{U}_i\right|^{-1/2} e^{-\frac{1}{2}\mathbf{r}^t\mathbf{U}_i^{-1}\mathbf{r}}
$$

$$
\times\left\{\begin{aligned}
&\frac{1}{2}\left[\mathbf{r}^t\mathbf{J}_i^{(\tau\upsilon)}\mathbf{r}-U^{-1}_{i,\tau\upsilon}\left(2-\delta_{\tau\upsilon}\right)\right]\Big[\left(\mathbf{r}^t\mathbf{U}_i^{-1}\mathbf{u}_\alpha\right)\left(\mathbf{r}^t\mathbf{U}_i^{-1}\mathbf{u}_\beta\right)\left(\mathbf{r}^t\mathbf{U}_i^{-1}\mathbf{u}_\gamma\right)\\
&-\left(\mathbf{r}^t\mathbf{U}_i^{-1}\mathbf{u}_\alpha\right)U^{-1}_{i,\beta\gamma}-\left(\mathbf{r}^t\mathbf{U}_i^{-1}\mathbf{u}_\beta\right)U^{-1}_{i,\alpha\gamma}-\left(\mathbf{r}^t\mathbf{U}_i^{-1}\mathbf{u}_\gamma\right)U^{-1}_{i,\alpha\beta}\Big]\\
&+\left(\mathbf{r}^t\mathbf{J}_i^{(\tau\upsilon)}\mathbf{u}_\alpha\right)\left(\mathbf{r}^t\mathbf{U}_i^{-1}\mathbf{u}_\beta\right)\left(\mathbf{r}^t\mathbf{U}_i^{-1}\mathbf{u}_\gamma\right)+\left(\mathbf{r}^t\mathbf{U}_i^{-1}\mathbf{u}_\alpha\right)\left(\mathbf{r}^t\mathbf{J}_i^{(\tau\upsilon)}\mathbf{u}_\beta\right)\left(\mathbf{r}^t\mathbf{U}_i^{-1}\mathbf{u}_\gamma\right)\\
&+\left(\mathbf{r}^t\mathbf{U}_i^{-1}\mathbf{u}_\alpha\right)\left(\mathbf{r}^t\mathbf{U}_i^{-1}\mathbf{u}_\beta\right)\left(\mathbf{r}^t\mathbf{J}_i^{(\tau\upsilon)}\mathbf{u}_\gamma\right)-\left(\mathbf{r}^t\mathbf{J}_i^{(\tau\upsilon)}\mathbf{u}_\alpha\right)U^{-1}_{i,\beta\gamma}-\left(\mathbf{r}^t\mathbf{J}_i^{(\tau\upsilon)}\mathbf{u}_\beta\right)U^{-1}_{i,\alpha\gamma}\\
&-\left(\mathbf{r}^t\mathbf{J}_i^{(\tau\upsilon)}\mathbf{u}_\gamma\right)U^{-1}_{i,\alpha\beta}-\left(\mathbf{r}^t\mathbf{U}_i^{-1}\mathbf{u}_\alpha\right)J^{(\tau\upsilon)}_{i,\beta\gamma}-\left(\mathbf{r}^t\mathbf{U}_i^{-1}\mathbf{u}_\beta\right)J^{(\tau\upsilon)}_{i,\alpha\gamma}-\left(\mathbf{r}^t\mathbf{U}_i^{-1}\mathbf{u}_\gamma\right)J^{(\tau\upsilon)}_{i,\alpha\beta}
\end{aligned}\right\}
$$

$$
\frac{\partial\left[\nabla_\alpha\nabla_\beta\nabla_\gamma\nabla_\delta\rho(\mathbf{r})\right]}{\partial U_{\tau\upsilon}} = (2\pi)^{-3/2}\sum_{i=1}^{n} a_i\left|\mathbf{U}_i\right|^{-1/2} e^{-\frac{1}{2}\mathbf{r}^t\mathbf{U}_i^{-1}\mathbf{r}}
$$

$$
\times\left\{\begin{aligned}
&\frac{1}{2}\left[\mathbf{r}^t\mathbf{J}_i^{(\tau\upsilon)}\mathbf{r}-U^{-1}_{i,\tau\upsilon}\left(2-\delta_{\tau\upsilon}\right)\right]\\
&\times\left[\begin{aligned}
&\left(\mathbf{r}^t\mathbf{U}_i^{-1}\mathbf{u}_\alpha\right)\left(\mathbf{r}^t\mathbf{U}_i^{-1}\mathbf{u}_\beta\right)\left(\mathbf{r}^t\mathbf{U}_i^{-1}\mathbf{u}_\gamma\right)\left(\mathbf{r}^t\mathbf{U}_i^{-1}\mathbf{u}_\delta\right)\\
&-\left(\mathbf{r}^t\mathbf{U}_i^{-1}\mathbf{u}_\alpha\right)\left(\mathbf{r}^t\mathbf{U}_i^{-1}\mathbf{u}_\beta\right)U^{-1}_{i,\gamma\delta}-\left(\mathbf{r}^t\mathbf{U}_i^{-1}\mathbf{u}_\alpha\right)\left(\mathbf{r}^t\mathbf{U}_i^{-1}\mathbf{u}_\gamma\right)U^{-1}_{i,\beta\delta}-\left(\mathbf{r}^t\mathbf{U}_i^{-1}\mathbf{u}_\alpha\right)\left(\mathbf{r}^t\mathbf{U}_i^{-1}\mathbf{u}_\delta\right)U^{-1}_{i,\beta\gamma}\\
&-\left(\mathbf{r}^t\mathbf{U}_i^{-1}\mathbf{u}_\beta\right)\left(\mathbf{r}^t\mathbf{U}_i^{-1}\mathbf{u}_\gamma\right)U^{-1}_{i,\alpha\delta}-\left(\mathbf{r}^t\mathbf{U}_i^{-1}\mathbf{u}_\beta\right)\left(\mathbf{r}^t\mathbf{U}_i^{-1}\mathbf{u}_\delta\right)U^{-1}_{i,\alpha\gamma}-\left(\mathbf{r}^t\mathbf{U}_i^{-1}\mathbf{u}_\gamma\right)\left(\mathbf{r}^t\mathbf{U}_i^{-1}\mathbf{u}_\delta\right)U^{-1}_{i,\alpha\beta}\\
&+U^{-1}_{i,\alpha\beta}U^{-1}_{i,\gamma\delta}+U^{-1}_{i,\alpha\gamma}U^{-1}_{i,\beta\delta}+U^{-1}_{i,\alpha\delta}U^{-1}_{i,\beta\gamma}
\end{aligned}\right]\\
&+\left(\mathbf{r}^t\mathbf{J}_i^{(\tau\upsilon)}\mathbf{u}_\alpha\right)\left(\mathbf{r}^t\mathbf{U}_i^{-1}\mathbf{u}_\beta\right)\left(\mathbf{r}^t\mathbf{U}_i^{-1}\mathbf{u}_\gamma\right)\left(\mathbf{r}^t\mathbf{U}_i^{-1}\mathbf{u}_\delta\right)+\left(\mathbf{r}^t\mathbf{U}_i^{-1}\mathbf{u}_\alpha\right)\left(\mathbf{r}^t\mathbf{J}_i^{(\tau\upsilon)}\mathbf{u}_\beta\right)\left(\mathbf{r}^t\mathbf{U}_i^{-1}\mathbf{u}_\gamma\right)\left(\mathbf{r}^t\mathbf{U}_i^{-1}\mathbf{u}_\delta\right)\\
&+\left(\mathbf{r}^t\mathbf{U}_i^{-1}\mathbf{u}_\alpha\right)\left(\mathbf{r}^t\mathbf{U}_i^{-1}\mathbf{u}_\beta\right)\left(\mathbf{r}^t\mathbf{J}_i^{(\tau\upsilon)}\mathbf{u}_\gamma\right)\left(\mathbf{r}^t\mathbf{U}_i^{-1}\mathbf{u}_\delta\right)+\left(\mathbf{r}^t\mathbf{U}_i^{-1}\mathbf{u}_\alpha\right)\left(\mathbf{r}^t\mathbf{U}_i^{-1}\mathbf{u}_\beta\right)\left(\mathbf{r}^t\mathbf{U}_i^{-1}\mathbf{u}_\gamma\right)\left(\mathbf{r}^t\mathbf{J}_i^{(\tau\upsilon)}\mathbf{u}_\delta\right)\\
&-\left(\mathbf{r}^t\mathbf{J}_i^{(\tau\upsilon)}\mathbf{u}_\alpha\right)\left[\left(\mathbf{r}^t\mathbf{U}_i^{-1}\mathbf{u}_\beta\right)U^{-1}_{i,\gamma\delta}+\left(\mathbf{r}^t\mathbf{U}_i^{-1}\mathbf{u}_\gamma\right)U^{-1}_{i,\beta\delta}+\left(\mathbf{r}^t\mathbf{U}_i^{-1}\mathbf{u}_\delta\right)U^{-1}_{i,\beta\gamma}\right]\\
&-\left(\mathbf{r}^t\mathbf{J}_i^{(\tau\upsilon)}\mathbf{u}_\beta\right)\left[\left(\mathbf{r}^t\mathbf{U}_i^{-1}\mathbf{u}_\alpha\right)U^{-1}_{i,\gamma\delta}+\left(\mathbf{r}^t\mathbf{U}_i^{-1}\mathbf{u}_\gamma\right)U^{-1}_{i,\alpha\delta}+\left(\mathbf{r}^t\mathbf{U}_i^{-1}\mathbf{u}_\delta\right)U^{-1}_{i,\alpha\gamma}\right]\\
&-\left(\mathbf{r}^t\mathbf{J}_i^{(\tau\upsilon)}\mathbf{u}_\gamma\right)\left[\left(\mathbf{r}^t\mathbf{U}_i^{-1}\mathbf{u}_\alpha\right)U^{-1}_{i,\beta\delta}+\left(\mathbf{r}^t\mathbf{U}_i^{-1}\mathbf{u}_\beta\right)U^{-1}_{i,\alpha\delta}+\left(\mathbf{r}^t\mathbf{U}_i^{-1}\mathbf{u}_\delta\right)U^{-1}_{i,\alpha\beta}\right]\\
&-\left(\mathbf{r}^t\mathbf{J}_i^{(\tau\upsilon)}\mathbf{u}_\delta\right)\left[\left(\mathbf{r}^t\mathbf{U}_i^{-1}\mathbf{u}_\alpha\right)U^{-1}_{i,\beta\gamma}+\left(\mathbf{r}^t\mathbf{U}_i^{-1}\mathbf{u}_\beta\right)U^{-1}_{i,\alpha\gamma}+\left(\mathbf{r}^t\mathbf{U}_i^{-1}\mathbf{u}_\gamma\right)U^{-1}_{i,\alpha\beta}\right]\\
&-\left(\mathbf{r}^t\mathbf{U}_i^{-1}\mathbf{u}_\alpha\right)\left(\mathbf{r}^t\mathbf{U}_i^{-1}\mathbf{u}_\beta\right)J^{(\tau\upsilon)}_{i,\gamma\delta}-\left(\mathbf{r}^t\mathbf{U}_i^{-1}\mathbf{u}_\alpha\right)\left(\mathbf{r}^t\mathbf{U}_i^{-1}\mathbf{u}_\gamma\right)J^{(\tau\upsilon)}_{i,\beta\delta}-\left(\mathbf{r}^t\mathbf{U}_i^{-1}\mathbf{u}_\alpha\right)\left(\mathbf{r}^t\mathbf{U}_i^{-1}\mathbf{u}_\delta\right)J^{(\tau\upsilon)}_{i,\beta\gamma}\\
&-\left(\mathbf{r}^t\mathbf{U}_i^{-1}\mathbf{u}_\beta\right)\left(\mathbf{r}^t\mathbf{U}_i^{-1}\mathbf{u}_\gamma\right)J^{(\tau\upsilon)}_{i,\alpha\delta}-\left(\mathbf{r}^t\mathbf{U}_i^{-1}\mathbf{u}_\beta\right)\left(\mathbf{r}^t\mathbf{U}_i^{-1}\mathbf{u}_\delta\right)J^{(\tau\upsilon)}_{i,\alpha\gamma}-\left(\mathbf{r}^t\mathbf{U}_i^{-1}\mathbf{u}_\gamma\right)\left(\mathbf{r}^t\mathbf{U}_i^{-1}\mathbf{u}_\delta\right)J^{(\tau\upsilon)}_{i,\alpha\beta}\\
&+J^{(\tau\upsilon)}_{i,\alpha\beta}U^{-1}_{i,\gamma\delta}+J^{(\tau\upsilon)}_{i,\alpha\gamma}U^{-1}_{i,\beta\delta}+J^{(\tau\upsilon)}_{i,\alpha\delta}U^{-1}_{i,\beta\gamma}\\
&+J^{(\tau\upsilon)}_{i,\beta\delta}U^{-1}_{i,\alpha\gamma}+J^{(\tau\upsilon)}_{i,\beta\gamma}U^{-1}_{i,\alpha\delta}+J^{(\tau\upsilon)}_{i,\gamma\delta}U^{-1}_{i,\alpha\beta}
\end{aligned}\right\}\quad(2)
$$

Derivative with respect to the Width of 3rd and 4th Order Cartesian Gaussian Tensors

$$\frac{\partial\left[\nabla_\alpha\nabla_\beta\nabla_\gamma\rho(\mathbf{r})\right]}{\partial\kappa}=-(2\pi)^{-3/2}\sum_{i=1}^{n}a_i\left|\mathbf{U}_i\right|^{-1/2}e^{-\frac{1}{2}\mathbf{r}^t\mathbf{U}_i^{-1}\mathbf{r}}$$

$$\times\left\{\begin{array}{l}\frac{1}{2}\left(-\mathbf{r}^t\mathbf{J}_i^{(\kappa)}\mathbf{r}-\left|\mathbf{U}_i\right|^{-1}\frac{\partial\left|\mathbf{U}_i\right|}{\partial\kappa}\right)\left[\left(\mathbf{r}^t\mathbf{U}_i^{-1}\mathbf{u}_\alpha\right)\left(\mathbf{r}^t\mathbf{U}_i^{-1}\mathbf{u}_\beta\right)\left(\mathbf{r}^t\mathbf{U}_i^{-1}\mathbf{u}_\gamma\right)\right.\\
\left.-\left(\mathbf{r}^t\mathbf{U}_i^{-1}\mathbf{u}_\alpha\right)U_{i,\beta\gamma}^{-1}-\left(\mathbf{r}^t\mathbf{U}_i^{-1}\mathbf{u}_\beta\right)U_{i,\alpha\gamma}^{-1}-\left(\mathbf{r}^t\mathbf{U}_i^{-1}\mathbf{u}_\gamma\right)U_{i,\alpha\beta}^{-1}\right]\\
+\left(\mathbf{r}^t\mathbf{J}_i^{(\kappa)}\mathbf{u}_\alpha\right)\left(\mathbf{r}^t\mathbf{U}_i^{-1}\mathbf{u}_\beta\right)\left(\mathbf{r}^t\mathbf{U}_i^{-1}\mathbf{u}_\gamma\right)+\left(\mathbf{r}^t\mathbf{U}_i^{-1}\mathbf{u}_\alpha\right)\left(\mathbf{r}^t\mathbf{J}_i^{(\kappa)}\mathbf{u}_\beta\right)\left(\mathbf{r}^t\mathbf{U}_i^{-1}\mathbf{u}_\gamma\right)\\
+\left(\mathbf{r}^t\mathbf{U}_i^{-1}\mathbf{u}_\alpha\right)\left(\mathbf{r}^t\mathbf{U}_i^{-1}\mathbf{u}_\beta\right)\left(\mathbf{r}^t\mathbf{J}_i^{(\kappa)}\mathbf{u}_\gamma\right)-\left(\mathbf{r}^t\mathbf{J}_i^{(\kappa)}\mathbf{u}_\alpha\right)U_{i,\beta\gamma}^{-1}-\left(\mathbf{r}^t\mathbf{J}_i^{(\kappa)}\mathbf{u}_\beta\right)U_{i,\alpha\gamma}^{-1}\\
-\left(\mathbf{r}^t\mathbf{J}_i^{(\kappa)}\mathbf{u}_\gamma\right)U_{i,\alpha\beta}^{-1}-\left(\mathbf{r}^t\mathbf{U}_i^{-1}\mathbf{u}_\alpha\right)J_{i,\beta\gamma}^{(\kappa)}-\left(\mathbf{r}^t\mathbf{U}_i^{-1}\mathbf{u}_\beta\right)J_{i,\alpha\gamma}^{(\kappa)}-\left(\mathbf{r}^t\mathbf{U}_i^{-1}\mathbf{u}_\gamma\right)J_{i,\alpha\beta}^{(\kappa)}\end{array}\right\}$$

$$\frac{\partial\left[\nabla_\alpha\nabla_\beta\nabla_\gamma\nabla_\delta\rho(\mathbf{r})\right]}{\partial\kappa}=(2\pi)^{-3/2}\sum_{i=1}^{n}a_i\left|\mathbf{U}_i\right|^{-1/2}e^{-\frac{1}{2}\mathbf{r}^t\mathbf{U}_i^{-1}\mathbf{r}}$$

$$\times\left\{\begin{array}{l}\frac{1}{2}\left(-\mathbf{r}^t\mathbf{J}_i^{(\kappa)}\mathbf{r}-\left|\mathbf{U}_i\right|^{-1}\frac{\partial\left|\mathbf{U}_i\right|}{\partial\kappa}\right)\\
\times\left[\begin{array}{l}\left(\mathbf{r}^t\mathbf{U}_i^{-1}\mathbf{u}_\alpha\right)\left(\mathbf{r}^t\mathbf{U}_i^{-1}\mathbf{u}_\beta\right)\left(\mathbf{r}^t\mathbf{U}_i^{-1}\mathbf{u}_\gamma\right)\left(\mathbf{r}^t\mathbf{U}_i^{-1}\mathbf{u}_\delta\right)\\
-\left(\mathbf{r}^t\mathbf{U}_i^{-1}\mathbf{u}_\alpha\right)\left(\mathbf{r}^t\mathbf{U}_i^{-1}\mathbf{u}_\beta\right)U_{i,\gamma\delta}^{-1}-\left(\mathbf{r}^t\mathbf{U}_i^{-1}\mathbf{u}_\alpha\right)\left(\mathbf{r}^t\mathbf{U}_i^{-1}\mathbf{u}_\gamma\right)U_{i,\beta\delta}^{-1}-\left(\mathbf{r}^t\mathbf{U}_i^{-1}\mathbf{u}_\alpha\right)\left(\mathbf{r}^t\mathbf{U}_i^{-1}\mathbf{u}_\delta\right)U_{i,\beta\gamma}^{-1}\\
-\left(\mathbf{r}^t\mathbf{U}_i^{-1}\mathbf{u}_\beta\right)\left(\mathbf{r}^t\mathbf{U}_i^{-1}\mathbf{u}_\gamma\right)U_{i,\alpha\delta}^{-1}-\left(\mathbf{r}^t\mathbf{U}_i^{-1}\mathbf{u}_\beta\right)\left(\mathbf{r}^t\mathbf{U}_i^{-1}\mathbf{u}_\delta\right)U_{i,\alpha\gamma}^{-1}-\left(\mathbf{r}^t\mathbf{U}_i^{-1}\mathbf{u}_\gamma\right)\left(\mathbf{r}^t\mathbf{U}_i^{-1}\mathbf{u}_\delta\right)U_{i,\alpha\beta}^{-1}\\
+U_{i,\alpha\beta}^{-1}U_{i,\gamma\delta}^{-1}+U_{i,\alpha\gamma}^{-1}U_{i,\beta\delta}^{-1}+U_{i,\alpha\delta}^{-1}U_{i,\beta\gamma}^{-1}\end{array}\right]\\
+\left(\mathbf{r}^t\mathbf{J}_i^{(\kappa)}\mathbf{u}_\alpha\right)\left(\mathbf{r}^t\mathbf{U}_i^{-1}\mathbf{u}_\beta\right)\left(\mathbf{r}^t\mathbf{U}_i^{-1}\mathbf{u}_\gamma\right)\left(\mathbf{r}^t\mathbf{U}_i^{-1}\mathbf{u}_\delta\right)+\left(\mathbf{r}^t\mathbf{U}_i^{-1}\mathbf{u}_\alpha\right)\left(\mathbf{r}^t\mathbf{J}_i^{(\kappa)}\mathbf{u}_\beta\right)\left(\mathbf{r}^t\mathbf{U}_i^{-1}\mathbf{u}_\gamma\right)\left(\mathbf{r}^t\mathbf{U}_i^{-1}\mathbf{u}_\delta\right)\\
+\left(\mathbf{r}^t\mathbf{U}_i^{-1}\mathbf{u}_\alpha\right)\left(\mathbf{r}^t\mathbf{U}_i^{-1}\mathbf{u}_\beta\right)\left(\mathbf{r}^t\mathbf{J}_i^{(\kappa)}\mathbf{u}_\gamma\right)\left(\mathbf{r}^t\mathbf{U}_i^{-1}\mathbf{u}_\delta\right)+\left(\mathbf{r}^t\mathbf{U}_i^{-1}\mathbf{u}_\alpha\right)\left(\mathbf{r}^t\mathbf{U}_i^{-1}\mathbf{u}_\beta\right)\left(\mathbf{r}^t\mathbf{U}_i^{-1}\mathbf{u}_\gamma\right)\left(\mathbf{r}^t\mathbf{J}_i^{(\kappa)}\mathbf{u}_\delta\right)\\
-\left(\mathbf{r}^t\mathbf{J}_i^{(\kappa)}\mathbf{u}_\alpha\right)\left[\left(\mathbf{r}^t\mathbf{U}_i^{-1}\mathbf{u}_\beta\right)U_{i,\gamma\delta}^{-1}+\left(\mathbf{r}^t\mathbf{U}_i^{-1}\mathbf{u}_\gamma\right)U_{i,\beta\delta}^{-1}+\left(\mathbf{r}^t\mathbf{U}_i^{-1}\mathbf{u}_\delta\right)U_{i,\beta\gamma}^{-1}\right]\\
-\left(\mathbf{r}^t\mathbf{J}_i^{(\kappa)}\mathbf{u}_\beta\right)\left[\left(\mathbf{r}^t\mathbf{U}_i^{-1}\mathbf{u}_\alpha\right)U_{i,\gamma\delta}^{-1}+\left(\mathbf{r}^t\mathbf{U}_i^{-1}\mathbf{u}_\gamma\right)U_{i,\alpha\delta}^{-1}+\left(\mathbf{r}^t\mathbf{U}_i^{-1}\mathbf{u}_\delta\right)U_{i,\alpha\gamma}^{-1}\right]\\
-\left(\mathbf{r}^t\mathbf{J}_i^{(\kappa)}\mathbf{u}_\gamma\right)\left[\left(\mathbf{r}^t\mathbf{U}_i^{-1}\mathbf{u}_\alpha\right)U_{i,\beta\delta}^{-1}+\left(\mathbf{r}^t\mathbf{U}_i^{-1}\mathbf{u}_\beta\right)U_{i,\alpha\delta}^{-1}+\left(\mathbf{r}^t\mathbf{U}_i^{-1}\mathbf{u}_\delta\right)U_{i,\alpha\beta}^{-1}\right]\\
-\left(\mathbf{r}^t\mathbf{J}_i^{(\kappa)}\mathbf{u}_\delta\right)\left[\left(\mathbf{r}^t\mathbf{U}_i^{-1}\mathbf{u}_\alpha\right)U_{i,\beta\gamma}^{-1}+\left(\mathbf{r}^t\mathbf{U}_i^{-1}\mathbf{u}_\beta\right)U_{i,\alpha\gamma}^{-1}+\left(\mathbf{r}^t\mathbf{U}_i^{-1}\mathbf{u}_\gamma\right)U_{i,\alpha\beta}^{-1}\right]\\
-\left(\mathbf{r}^t\mathbf{U}_i^{-1}\mathbf{u}_\alpha\right)\left(\mathbf{r}^t\mathbf{U}_i^{-1}\mathbf{u}_\beta\right)J_{i,\gamma\delta}^{(\kappa)}-\left(\mathbf{r}^t\mathbf{U}_i^{-1}\mathbf{u}_\alpha\right)\left(\mathbf{r}^t\mathbf{U}_i^{-1}\mathbf{u}_\gamma\right)J_{i,\beta\delta}^{(\kappa)}-\left(\mathbf{r}^t\mathbf{U}_i^{-1}\mathbf{u}_\alpha\right)\left(\mathbf{r}^t\mathbf{U}_i^{-1}\mathbf{u}_\delta\right)J_{i,\beta\gamma}^{(\kappa)}\\
-\left(\mathbf{r}^t\mathbf{U}_i^{-1}\mathbf{u}_\beta\right)\left(\mathbf{r}^t\mathbf{U}_i^{-1}\mathbf{u}_\gamma\right)J_{i,\alpha\delta}^{(\kappa)}-\left(\mathbf{r}^t\mathbf{U}_i^{-1}\mathbf{u}_\beta\right)\left(\mathbf{r}^t\mathbf{U}_i^{-1}\mathbf{u}_\delta\right)J_{i,\alpha\gamma}^{(\kappa)}-\left(\mathbf{r}^t\mathbf{U}_i^{-1}\mathbf{u}_\gamma\right)\left(\mathbf{r}^t\mathbf{U}_i^{-1}\mathbf{u}_\delta\right)J_{i,\alpha\beta}^{(\kappa)}\\
+J_{i,\alpha\beta}^{(\kappa)}U_{i,\gamma\delta}^{-1}+J_{i,\alpha\gamma}^{(\kappa)}U_{i,\beta\delta}^{-1}+J_{i,\alpha\delta}^{(\kappa)}U_{i,\beta\gamma}^{-1}\\
+J_{i,\beta\delta}^{(\kappa)}U_{i,\alpha\gamma}^{-1}+J_{i,\beta\gamma}^{(\kappa)}U_{i,\alpha\delta}^{-1}+J_{i,\gamma\delta}^{(\kappa)}U_{i,\alpha\beta}^{-1}\end{array}\right\}\quad(3)$$
