# Supplementary material for: Polarizable atomic multipole X-ray refinement: application to peptide crystals
Source: Acta Crystallogr D Biol Crystallogr. 2009 Aug 14;65(Pt 9):952–65. doi: 10.1107/S0907444909022707 (PMC2733883; doi:10.1107/S0907444909022707)
Supplement: Supplementary file 2 [file d-65-00952-sup2.pdf]

Supplementary Information (Mathematica Notebook) for:

Polarizable Atomic Multipole X-Ray Refinement: Application to Peptide Crystals

Michael J. Schnieders, Timothy D. Fenn, Vijay S. Pande and Axel T. Brunger

Department of Chemistry, Stanford CA, 94305, USA;
Department of Molecular and Cellular Physiology, Stanford CA, 94305, USA;
Howard Hughes Medical Institute, USA.

E-mail: schnied@stanford.edu

Define a term of the spherical and anisotropic scattering density given in Eq. (6).

```
(* Position vector relative to the atomic center. *)
r = {x, y, z};
(* ADP matrix *)
U = {{Subscript[u, 1, 1], Subscript[u, 1, 2], Subscript[u, 1, 3]},
   {Subscript[u, 1, 2], Subscript[u, 2, 2], Subscript[u, 2, 3]},
   {Subscript[u, 1, 3], Subscript[u, 2, 3], Subscript[u, 3, 3]}};
Ui = U + (Subscript[b, i] / (8 * π^2 * κ^2) + Subscript[u, add]) * IdentityMatrix[3];
detUi = Det[Ui];
invUi = Inverse[Ui];
(* One term of the spherical, anisotropic scattering factor given in Eq. (6) *)
ρi = (2 * π) ^ (-3 / 2) * Subscript[a, i] * detUi ^ (-1 / 2) * Exp[-r.invUi.r / 2];
```

Construct and plot example aspherical and anisotropic scattering densities as given in Eq. (16).

```
ρix = D[ρi, x];
ρiy = D[ρi, y];
ρiz = D[ρi, z];
ρixx = D[ρix, x];
ρiyy = D[ρiy, y];
ρizz = D[ρiz, z];
ρixy = D[ρix, y];
ρiyz = D[ρiy, z];
ρixz = D[ρix, z];

(* Varying the multipole coefficients below while observing the
  isosurface plots provides intuition into the characteristics of the
  aspherical and anisotropic Cartesian Gaussian multipole basis set. The
  default coefficients produce an axial quadrupole along the Z-axis. *)
q = 0;
dx = 0;
dy = 0;
dz = 0;
qxx = -1;
qyy = -1;
qzz = 2;
qxy = 0;
qxz = 0;
qyz = 0;

(* Make the quadrupole traceless *)
qave = (qxx + qyy + qzz) / 3;
qxx = qxx - qave;
qyy = qyy - qave;
qzz = qzz - qave;

ρadp = -q * ρi + dx * ρix + dy * ρix + dz * ρiz -
   1 / 3 (qxx * ρixx + qyy * ρiyy + qzz * ρizz + 2 * (qxy * ρixy + qxz * ρixz + qyz * ρiyz));

(* Apply an isotropic ADP. *)
ρplot =
  FullSimplify[ρadp /. Subscript[b, i] → 1 /. Subscript[a, i] → 1 /. Subscript[u, 1, 1] → 1 /.
          Subscript[u, 1, 2] → 0 /. Subscript[u, 1, 3] → 0 /. Subscript[u, 2, 2] → 1 /.
        Subscript[u, 2, 3] → 0 /. Subscript[u, 3, 3] → 1 /. κ → 1 /. Subscript[u, add] → 0];

ContourPlot3D[ρplot, {x, -3, 3}, {y, -3, 3}, {z, -3, 3},
 Contours → {{-0.01, {Red, Specularity[White, 50]}}, {0.01, {Blue, Specularity[White, 50]}}},
 Mesh → None, Boxed → False, Axes → False, ColorFunctionScaling → False,
 Lighting → {{"Directional", RGBColor[1, 1, 1], {{1, 0, 1}, {0, 0, 0}}}}]

(* Apply an anisotropic ADP (uxx = 1.5, uyy = 1, uzz = 1)
  to thermally smear the density along the X-axis. *)
ρplot = FullSimplify[
   ρadp /. Subscript[b, i] → 1 /. Subscript[a, i] → 1 /. Subscript[u, 1, 1] → 1.5 /.
          Subscript[u, 1, 2] → 0 /. Subscript[u, 1, 3] → 0 /. Subscript[u, 2, 2] → 1 /.
        Subscript[u, 2, 3] → 0 /. Subscript[u, 3, 3] → 1 /. κ → 1 /. Subscript[u, add] → 0];

ContourPlot3D[ρplot, {x, -3, 3}, {y, -3, 3}, {z, -3, 3},
 Contours → {{-0.01, {Red, Specularity[White, 50]}}, {0.01, {Blue, Specularity[White, 50]}}},
 Mesh → None, Boxed → False, Axes → False, ColorFunctionScaling → False,
 Lighting → {{"Directional", RGBColor[1, 1, 1], {{1, 0, 1}, {0, 0, 0}}}},
 ContourStyle → Directive[Specularity[White, 50]]]
```

Verify analytic forms of the Cartesian Gaussian multipole tensors from Eq. (18).

```
ρixxx = D[ρixx, x];
ρixxy = D[ρixx, y];
ρixyz = D[ρixy, z];
ρixxxx = D[ρixxx, x];
ρixxxxx = D[ρixxxx, x];

 (* x unit vector *)
ux = {1, 0, 0};
(* y unit vector *)
uy = {0, 1, 0};
(* z unit vector *)
uz = {0, 0, 1};

tx = -ρi * (r.invUi.ux);
txx = ρi * ((r.invUi.ux) ^2 - invUi[[1, 1]]);
txy = ρi * ((r.invUi.ux) * (r.invUi.uy) - invUi[[1, 2]]);
txxx = -ρi * ((r.invUi.ux) ^3 - 3 * (r.invUi.ux) * invUi[[1, 1]]);
txxy = -ρi * ((r.invUi.ux) ^2 * (r.invUi.uy) -
      2 * (r.invUi.ux) * invUi[[1, 2]] - (r.invUi.uy) * invUi[[1, 1]] );
txyz = -ρi * ((r.invUi.ux) * (r.invUi.uy) * (r.invUi.uz) - (r.invUi.ux) * invUi[[2, 3]] -
      (r.invUi.uy) * invUi[[1, 3]] - (r.invUi.uz) * invUi[[1, 2]] );
txxxx = ρi * ((r.invUi.ux) ^4 - 6 * (r.invUi.ux) ^2 * invUi[[1, 1]] + 3 * invUi[[1, 1]] ^2);
txxxxx = -ρi *
    ((r.invUi.ux) ^5 - 10 * (r.invUi.ux) ^3 * invUi[[1, 1]] + 15 * (r.invUi.ux) * invUi[[1, 1]] ^2);

FullSimplify[ρix - tx]
FullSimplify[ρixx - txx];
FullSimplify[ρixy - txy];
FullSimplify[ρixxx - txxx];
FullSimplify[ρixxy - txxy];
FullSimplify[ρixyz - txyz];
FullSimplify[ρixxxx - txxxx];
FullSimplify[ρixxxxx - txxxxx]
```

Verify analytic derivatives of the Cartesian Gaussian multipole tensors with respect to ADPs given in Eq. (28).

```
(* The matrices Jxx and Jyy are constructed
 for convenience and are examples of Eq. (26). *)
Jxx = Simplify[ -invUi.D[Ui, Subscript[u, 1, 1]].invUi];
Jxy = Simplify[-invUi.D[Ui, Subscript[u, 1, 2]].invUi];
Simplify[D[invUi, Subscript[u, 1, 1]] - Jxx]
Simplify[D[invUi, Subscript[u, 1, 2]] - Jxy]

ρiU11 = D[ρi, Subscript[u, 1, 1]];
ρiU12 = D[ρi, Subscript[u, 1, 2]];
ρixU11 = D[ρix, Subscript[u, 1, 1]];
ρixU12 = D[ρix, Subscript[u, 1, 2]];
ρixxU11 = D[ρixx, Subscript[u, 1, 1]];
ρixxU12 = D[ρixx, Subscript[u, 1, 2]];
ρixyU11 = D[ρixy, Subscript[u, 1, 1]];
ρixyU12 = D[ρixy, Subscript[u, 1, 2]];
ρixxxU11 = D[ρixxx, Subscript[u, 1, 1]];
ρixxxU12 = D[ρixxx, Subscript[u, 1, 2]];

tU11 = ρi * (1 / 2 * (-r.Jxx.r - invUi[[1, 1]]));
tU12 = ρi * (1 / 2 * (-r.Jxy.r - 2 * invUi[[1, 2]]));
txU11 = -ρi * (1 / 2 * (-r.Jxx.r - invUi[[1, 1]]) * r.invUi.ux + r.Jxx.ux);
txU12 = -ρi * (1 / 2 * (-r.Jxy.r - 2 * invUi[[1, 2]]) * r.invUi.ux + r.Jxy.ux);
txxU11 = ρi * (1 / 2 * (-r.Jxx.r - invUi[[1, 1]]) * ((r.invUi.ux) ^ 2 - invUi[[1, 1]]) +
      2 * (r.Jxx.ux) * (r.invUi.ux) - Jxx[[1, 1]]);
txxU12 = ρi * (1 / 2 * (-r.Jxy.r - 2 * invUi[[1, 2]]) * ((r.invUi.ux) ^ 2 - invUi[[1, 1]]) +
      2 * (r.Jxy.ux) * (r.invUi.ux) - Jxy[[1, 1]]);
txyU11 = ρi * (1 / 2 * (-r.Jxx.r - invUi[[1, 1]]) * ((r.invUi.ux) * (r.invUi.uy) - invUi[[1, 2]]) +
      (r.Jxx.ux) * (r.invUi.uy) + (r.Jxx.uy) * (r.invUi.ux) - Jxx[[1, 2]]);
txyU12 = ρi * (1 / 2 * (-r.Jxy.r - 2 * invUi[[1, 2]]) * ((r.invUi.ux) * (r.invUi.uy) - invUi[[1, 2]]) +
      (r.Jxy.ux) * (r.invUi.uy) + (r.Jxy.uy) * (r.invUi.ux) - Jxy[[1, 2]]);
txxxU11 = -ρi * (1 / 2 * (-r.Jxx.r - invUi[[1, 1]]) *
       ((r.invUi.ux) ^ 3 - 3 * (r.invUi.ux) * invUi[[1, 1]]) + 3 * (r.Jxx.ux) * (r.invUi.ux) ^ 2 -
      3 * (r.invUi.ux) * Jxx[[1, 1]] - 3 * (r.Jxx.ux) * invUi[[1, 1]]);
txxxU12 = -ρi * (1 / 2 * (-r.Jxy.r - 2 * invUi[[1, 2]]) *
       ((r.invUi.ux) ^ 3 - 3 * (r.invUi.ux) * invUi[[1, 1]]) + 3 * (r.Jxy.ux) * (r.invUi.ux) ^ 2 -
      3 * (r.invUi.ux) * Jxy[[1, 1]] - 3 * (r.Jxy.ux) * invUi[[1, 1]]);

FullSimplify[ρiU11 - tU11]
FullSimplify[ρiU12 - tU12];
FullSimplify[ρixU11 - txU11];
FullSimplify[ρixU12 - txU12];
FullSimplify[ρixxU11 - txxU11];
FullSimplify[ρixxU12 - txxU12];
FullSimplify[ρixyU11 - txyU11];
FullSimplify[ρixyU12 - txyU12];
FullSimplify[ρixxxU11 - txxxU11];
FullSimplify[ρixxxU12 - txxxU12]
```

Verify analytic derivatives of the Cartesian Gaussian multipole tensors with respect to κ given in Eq. (34).

```
(* Intermediate term given in Eq. (30). *)
Uiκ = -3 * Subscript[b, i]^3 / (256 * π^6 * κ^7) -
   Subscript[b, i]^2 * (Subscript[u, 1, 1] + Subscript[u, 2, 2] +
       Subscript[u, 3, 3] + 3 * Subscript[u, add]) / (16 * π^4 * κ^5) +
   Subscript[b, i] * (Subscript[u, 1, 2]^2 + Subscript[u, 1, 3]^2 + Subscript[u, 2, 3]^2 -
       Subscript[u, 1, 1] * Subscript[u, 2, 2] - Subscript[u, 1, 1] * Subscript[u, 3, 3] -
       Subscript[u, 2, 2] * Subscript[u, 3, 3] - 2 * Subscript[u, add] * (Subscript[u, 1, 1] +
          Subscript[u, 2, 2] + Subscript[u, 3, 3]) - 3 * Subscript[u, add]^2) / (4 * π^2 * κ^3);
FullSimplify[Uiκ - D[detUi, κ]]
(* Matrix Jκ is constructed for convenience as in Eq. (33). *)
Jκ = Simplify[Subscript[b, i] / (4 * π^2 * κ^3) * invUi.invUi];
FullSimplify[Jκ - D[invUi, κ]]
ρiκ = D[ρi, κ];
ρixκ = D[ρix, κ];
ρixxκ = D[ρixx, κ];
ρixyκ = D[ρixy, κ];
ρixxxκ = D[ρixxx, κ];

tκ = ρi * (1 / 2) * (-r.Jκ.r - Uiκ / detUi);
txκ = -ρi * ((1 / 2) * (-r.Jκ.r - Uiκ / detUi) * (r.invUi.ux) + r.Jκ.ux);
txxκ = ρi * ((1 / 2) * (-r.Jκ.r - Uiκ / detUi) * ((r.invUi.ux)^2 - invUi[[1, 1]]) +
      2 * (r.Jκ.ux) * (r.invUi.ux) - Jκ[[1, 1]]);
txyκ = ρi * ((1 / 2) * (-r.Jκ.r - Uiκ / detUi) * ((r.invUi.ux) * (r.invUi.uy) - invUi[[1, 2]]) +
      (r.Jκ.ux) * (r.invUi.uy) + (r.Jκ.uy) * (r.invUi.ux) - Jκ[[1, 2]]);
txxxκ = -ρi * ((1 / 2) * (-r.Jκ.r - Uiκ / detUi) * ((r.invUi.ux)^3 - 3 * (r.invUi.ux) * invUi[[1, 1]]) +
      3 * (r.Jκ.ux) * (r.invUi.ux)^2 -
      3 * (r.invUi.ux) * Jκ[[1, 1]] - 3 * (r.Jκ.ux) * invUi[[1, 1]]);

FullSimplify[ρiκ - tκ]
FullSimplify[ρixκ - txκ];
FullSimplify[ρixxκ - txxκ];
FullSimplify[ρixyκ - txyκ];
FullSimplify[ρixxxκ - txxxκ]
```
